# Phytochemical Characterization of *Citrus*-Based Products Supporting Their Antioxidant Effect and Sensory Quality

**DOI:** 10.3390/foods11111550

**Published:** 2022-05-25

**Authors:** Ylenia Pieracci, Laura Pistelli, Massimiliano Cecchi, Luisa Pistelli, Marinella De Leo

**Affiliations:** 1 Department of Pharmacy, University of Pisa, Via Bonanno Pisano 33, 56126 Pisa, Italy; ylenia.pieracci@phd.unipi.it (Y.P.); luisa.pistelli@unipi.it (L.P.); 2 Department of Agriculture Food Environment, University of Pisa, Via del Borghetto 80, 56124 Pisa, Italy; laura.pistelli@unipi.it; 3 Interdepartmental Research Center, Nutraceuticals and Food for Health, University of Pisa, Via del Borghetto 80, 56124 Pisa, Italy; 4 Centre for Instrumentation Sharing, University of Pisa, Lungarno Pacinotti 43, 56126 Pisa, Italy; 5 Scientific Consultant, Via Novelli detto Yambo 19, 56124 Pisa, Italy; phdcecchi@gmail.com

**Keywords:** *Citrus* fruit, marmalade, phenols, limonoids, volatiles, antioxidant, sensorial analysis, food waste, LC-MS/Orbitrap, HS-SPME

## Abstract

The increasing attention on the impact of food on human and environmental health has led to a greater awareness about nutrition, food processing, and food waste. In this perspective, the present work deals with the investigation of the chemical non-volatile and volatile profiles of two *Citrus*-based products, produced through a conscious process, using *Citrus* peels as natural gelling agents. Moreover, the total polyphenol content (TPC) and the antioxidant properties were evaluated, as well as their sensorial properties. Chemical and antioxidant results were compared with those of *Citrus* fresh fruits (*C. reticulata*, *C. sinensis*, and *C. limon*). Concerning the non-volatile fingerprint, the two samples showed a very similar composition, characterized by flavanones (naringenin, hesperetin, and eriodyctiol *O*-glycosides), flavones (diosmetin and apigenin *C*-glucosides), and limonoids (limonin, nomilinic acid, and its glucoside). The amount of both flavonoids and limonoids was higher in the Lemon product than in the Mixed Citrus one, as well as the TPC and the antioxidant activity. The aroma composition of the two samples was characterized by monoterpene hydrocarbons as the main chemical class, mainly represented by limonene. The sensorial analysis, finally, evidenced a good quality of both the products. These results showed that the most representative components of *Citrus* fruits persist even after the transformation process, and the aroma and sensorial properties endow an added value to *Citrus* preparations.

## 1. Introduction

In recent decades, the attention on nutrition and its impact on health and economic growth is dramatically increased. In particular, the global demand for fruits and vegetables is rapidly growing, mainly due to the benefits on human health provided by their intake [1].

Recently, extensive scientific reviews have provided evidence that a high consumption of fruit and vegetables is associated with the prevention of chronic noncommunicable diseases (NCDs), such as cardiovascular diseases, cancer, diabetes, as well as neurodegenerative disorders [2,3,4,5,6]. NCDs are responsible for 71% of death globally, thus, as a part of 2030 Agenda for Sustainable Development, the reduction by one-third in premature mortality from NCDs through prevention and management represents the major challenge for sustainable development [7]. Unhealthy diet and lifestyle may play a role in increasing metabolic risk factors (e.g., blood pressure, blood glucose, and lipids), thus the World Health Organization (WHO) recommends an intake of at least 400 g per day of fruits and vegetables to reduce the risk of certain NCDs and to promote healthy nutrition [8].

Fruits and vegetables are a good source of nutrients, such as fibers, minerals, and vitamins. In addition, non-nutrients compounds, also called phytochemicals, are secondary plant metabolites, including polyphenols and triterpenoids, extensively known for many biological activities and benefits on human health [9,10].

A large amount of scientific literature is focused on health-promoting effects of polyphenols and related antioxidant agents (hydroxycinnamic acids, flavonoids, stilbenes, lignans) contained in fruits and vegetables in preventing the occurrence of degenerative diseases. Polyphenols were shown to exert antioxidant and free radical scavenging activities, as well as anti-inflammatory, antimicrobial, antiproliferative, cardioprotective, neuroprotective, and hepatoprotective properties [11,12,13,14].

Fruit and vegetables were classified in major groups, based on nutrient and phytochemical levels, to help consumer choice and nutritional professionals, and in subgroups considering botanical properties, total antioxidant capacity (TAC), and average levels of components. Among subgroups, the “*Citrus* fruit family” includes clementine, grapefruit (white and pink), kumquat, lemon, lime, orange, and tangerine. In relation to food components, *Citrus* fruit subgroup was found to be the highest in flavanones and the second highest in lycopene and flavones, providing at least 25% Dietary Recommended Intakes (DRI) for vitamin C/100 g. In addition, TAC ranged from 1000 to 3000 μmol Trolox Equivalent (TE)/100 g [10,15].

Fruits of the genus *Citrus* (Rutaceae family) are cultivated worldwide, and the market demand is constantly growing globally. The total production of *Citrus* fruit in the world reached 43,755.6 thousand tons in 2019, against 126,250.1 in 2011 [16]. *Citrus* fruits are considered as a part of the human diet, due to the their low energy and fat content, good amount of macronutrients (carbohydrates, dietary fiber, organic acids, vitamins), minerals, and bioactive phytochemicals, such as carotenoids (e.g., β-cryptoxanthin), flavonoids, limonoids, and essential oil [17]. Flavonoids are present in *Citrus* fruits as flavanones (e.g., hesperetin, naringenin, eriodictyol, isosakuranetin), flavones (luteolin, apigenin, diosmetin, chrysoeriol), and flavonols (quercetin, kaempferol), in the form of *O*-glycosides or *C*-glycosides [18,19]. The beneficial effects of *Citrus* flavonoids were investigated in a large number of studies reporting many pharmacological activities and health-promoting properties, such as antioxidant, anti-inflammatory, anticarcinogenic, cardiovascular protection, and beneficial effects on metabolic diseases [20,21,22]. Limonoids are oxygenated triterpenoids occurring in *Citrus* fruits both as aglycones and glycosides, particularly in by-products of juice production, such as seeds and peels [23]. Limonoids are considered responsible for the delayed bitterness of *Citrus* juice, especially when they occur in the form of aglycone, while the glycosylation confers a more pleasant taste to *Citrus* juice and products [24]. Recently, *Citrus* limonoids have been extensively studied for their potential biological activities including antioxidant, antimicrobial, insecticidal, anticancer, antidiabetic, and hypocholesterolemic effects [23,24]. Thus, both flavonoids and limonoids occurring in *Citrus* fruits contribute, separately or synergistically, to beneficial effects of *Citrus* fruits and related products on human health and well-being.

Concurrently with the large demand of fruits and vegetables as sources of health-promoting agents, the food industry has evolved and oriented to the development of novel and functional foods. Meanwhile, a great attention has been addressed to the effects of food processing on chemical composition, properties, quality, and safety of foods [1].

*Citrus* fruits are consumed either fresh or as processed products, such as juice, marmalade, jelly, and dehydrated products [25]. About a third of *Citrus* fruit worldwide is consumed after processing [16]. The high content in water make *Citrus* fruit highly perishable, thus marmalade and jam are the most common processing methods used for their preservation, prolonging shelf life and their off-season utilization [25]. In the last years, the nutraceutical properties of products coming from fruit processing have attracted attention, with particular regard to their chemical content in terms of biologically active substances as well as their antioxidant capacity [26,27,28,29]. Few previous studies are reported in the literature on *Citrus* jam/marmalade (orange and mandarin) that highlight the presence of bioactive components having good antioxidant capacity, even though in lower amount with respect to fresh fruits [30,31,32].

As a result of *Citrus* fruit processing, a large amount of waste (about 50% of whole fruit) is produced with a high impact on environmental aspects, thus recently *Citrus* byproducts have been investigated as source of bioactive molecules to be used as food, beverage, pharmaceutical, and cosmetic ingredients. Peels, membranes, and seeds constitute the solid residue of *Citrus* waste, thus their reuse can contribute to reduce the impact of waste/by-products [25]. In a recent study, the addition of orange peel in orange jam formulation resulted in a good strategy to improve the phenol content and the antioxidant capacity [33], as well as to replace pectin as a gelling agent [34]. As other examples, in our previous collaboration studies, olive oil and bread were fortified with *Citrus* peel/leaf and albedo, respectively, obtaining products with improved organoleptic properties and enriched with some *Citrus* phytochemicals [35,36,37,38].

In continuing our investigations on the chemical profile and health-promoting properties of several *Citrus* fruits typical of the Mediterranean area, such as bergamot (*Citrus × bergamia* Risso & Poiteau), Italian chinotto (*Citrus × myrtifolia* (Ker Gawl.) Raf.), Sardinian Pompia (*Citrus limon* var. *pompia* Camarda var. *nova*) and mandarin (*Citrus reticulata* Blanco) [39,40,41,42,43], the attention was herein focused on commercial *Citrus*-based products (Coctura^®^) obtained from lemons, oranges, and mandarins of selected cultivars provided by local farms located in Sicily (Italy) and processed as marmalades, with some modifications, such as the addition of *Citrus* peels in an optimized amount. The reduction of fruit waste as well as the valorization of local species as contribution to the biodiversity preservation are two important concepts at the basis of the production of *Citrus*-based Coctura^®^ products.

The aim of the present work was to investigate in depth the chemical fingerprint of two *Citrus*-based products with respect to flavonoids and triterpenoids, as well as the volatile organic compounds, to evaluate the preservation of health-promoting phytochemicals typical of *Citrus* fruits. The chemical composition of the final products was compared with that of fresh *Citrus* fruits utilized for their preparation. The quantitative analysis of the major identified compounds was also performed. Highly sensitive analytical techniques were used, such as ultra-high performance liquid chromatography coupled to a high resolution Orbitrap-based mass spectrometer (UHPLC-HR-MS/Orbitrap) and gas-chromatography coupled to MS (GC-MS) for non-volatile and volatile compounds, respectively. In addition, the total polyphenol content (TPC) and the antioxidant properties were evaluated to investigate the nutraceutical properties of the final products. Taking into account how the acceptability of food products is important among consumers, a sensorial analysis was finally performed.

## 2. Materials and Method

### 2.1. Chemicals and Reagents

Analytical grade methanol and *n*-butanol used for extract preparation were purchased from Merck (Darmstadt, Germany). UHPLC grade methanol, formic acid, and water were supplied from Romil-Deltek (Pozzuoli, Italy). DPPH (2,2-diphenyl-1-picrylhydrazyl), ABTS (2,2′-azino-bis(3-ethylbenzothiazoline-6-sulfonic acid) and Folin–Ciocalteu reagent were purchased by Merck (Darmstadt, Germany). Limonin standard (99.8% purity) was obtained from DBA Italia s.r.l. (Milano, Italy). Hesperidin (97% purity) and vitexin (≥95% purity) were purchased from Merck (Darmstadt, Germany).

### 2.2. Citrus Fruits and Commercial Products

#### 2.2.1. Fruit Origin and Product Composition

Commercial Coctura^®^ products and *Citrus* fruits were kindly supplied by the company “TERRA AQUA s.r.l.” located in Verona (Italy).

“Coctura^®^ Mixed Citrus” is a gastronomic specialty based on *Citrus* fruits cultivated by local farms in the territory of Santo Stefano di Briga, an ancient village located in Messina province (Sicily, Italy), crossed by the torrent Santo Stefano. The used *Citrus* fruits are sweet oranges (*Citrus sinensis* (L.) Osbeck), mandarins (*Citrus reticulata* Blanco cv. Avana), and lemons (*Citrus limon* (L.) Osbeck cv. Femminello Santa Teresa). “Coctura^®^ Lemon” is based only on Femminello Santa Teresa lemons. All fresh fruits were collected in February 2021; the pulps and peels were separated and stored at −20 °C until extract preparations.

The composition of both the commercial products is shown in Table 1.

#### 2.2.2. Product Preparation

The fruit products were prepared following the process of preparation of marmalade/jam, according to the Italian Legislative Decree n° 50/2004 and the European Union Council Directive 2001/113EC, while food information to consumers about the origin of fruits agrees with the Regulation (EU) 2018/775.

The *Citrus* fruits were collected from plants at ripening stage, then selected for quality and processed, separating pulp, seeds, and peels. All the operations were made manually. The selected fruit material was successively mixed with cane sugar and placed in a steel ball for cooking under vacuum (<1 bar) at T < 65 °C to preserve the quality of fruits and organoleptic characteristics. The cooked material was then concentrated in sugars, stirred, and transferred into filled glass jars while still warm, then capped and subjected to pasteurization at T = 98 °C for no more than 15 min. All jars were left at room temperature for at least 24 h for cooling. No additional pectin, flavours, or preservative agents were added.

### 2.3. Preparation of Extracts

*Citrus* pulps, deprived of seeds, were lyophilized (Modulyo, Pirani 501, Edwards, UK), while peels were dried in an oven at T = 39 °C. Mixed Citrus and Lemon products, as well as the dried fruits peels and pulps, were subjected to an extraction process to recover the potential bioactive molecules.

Each sample (15 g of *Citrus* products, fruits peels, and fruits pulps) was extracted with methanol by ultrasound-assisted extraction using a Labsonic LBS2 ultrasonic bath (Falc Instruments s.r.l., Treviglio, Italy) for 15 min (T = 20 °C, 59 kHz), then centrifuged (4000 rpm for 10 min). The supernatants were recovered, and the solvent removed under vacuum to obtain dry methanol extracts as reported in Table 2.

For UHPLC-MS analyses, the obtained extracts were successively partitioned between *n*-butanol and water to remove the sugar content. After mixing for 5 min, the organic and aqueous phases were separated by centrifugation (4000 rpm) for 10 min. The *n*-butanol solutions were dried under vacuum to obtain dry extracts (Table 2), which were injected into the LC-MS system.

### 2.4. Total Polyphenol Content

Total polyphenol content (TPC) was assessed by a modified protocol of the Folin–Ciocalteu method [44]. The analysis was performed in triplicates. The *n*-butanolic extracts of all samples (0.150 g each) were diluted with 2 mL of MeOH 70%. The diluted solution (50 μL) was determined as already described. The incubation was performed at 40 °C for 30 min, then the absorbance was determined at 765 nm in a UV-1800 spectrophotometer (Shimadzu Corporation, Kyoto, Japan). TPC were referred as gallic acid as standard and expressed as mg GAE/100 g of original product (mg gallic acid equivalents/100 g).

### 2.5. Antioxidant Activity

The antioxidant activity of the fruits pulps and peels and of the two *Citrus*-based products (Mixed Citrus and Lemon) was assessed in triplicates by free radical scavenging DPPH (2,2-diphenyl-1-picrylhydrazyl radical) activity and ABTS (2,2-azino-bis(ethylbenzene-thiazoline-6-sulfonic acid) assays.

For the DPPH assay, 10, 20, 40, and 60 μL of the methanolic solutions of the samples were added to a DPPH solution to reach a final volume of 1 mL. After 30 min of incubation at room temperature in the dark, the blenching of DPPH was measured at 517 nm. The results were expressed as IC_50_ value, calculated from the regression line relating the DPPH inhibition ratio and the sample volume, using the interpolation method. The inhibition ratio (I %) was calculated as [(Abs0 − Abs1/Abs0) × 100], where Abs0 is the absorbance of the DPPH and Abs1 is the absorbance of the sample, according to Brand-Williams et al. [45]. The ABTS assay was assessed according to the Re et al. method [46], using 20 μL of the fruit pulps and marmalades’ methanolic solutions and 5 μL of the fruit peels’ methanolic solutions. The oxidization of the ABTS with potassium persulfate produced the green/blue radical cation, ABTS^+^. ABTS^+^ was reduced, in turn, by the presence of hydrogen-donating antioxidants, determining a decolorization of the green/blue chromogen, determined spectrophotometrically at 734 nm. Trolox was used as control (2.5 mM). The results were expressed as mM of Trolox equivalents per 100 g of fresh product (fruits and marmalades).

### 2.6. Chemical Characterization by UHPLC-ESI-HR-MS/Orbitrap Analysis

The chemical content of the fruits used for the manufacturing of the analysed products and of the two *Citrus*-based marmalades was investigated by means of ultra-high-performance liquid chromatography (UHPLC, Vanquish Flex Binary pump) coupled to an electrospray ionization (ESI) high resolution-mass spectrometer (HR-MS) Q Exactive Plus MS Orbitrap-based FT-MS system (Thermo Fischer Scientific Inc., Bremem, Germany). For UHPLC-MS analyses, the *n*-butanol extracts were dissolved in methanol (2 mg/mL) and centrifuged (4000 rpm) for 10 min, then the supernatants were injected (5 µL injection volume) on a C-18 Kinetex^®^ Biphenyl column (100 × 2.1 mm, 2.6 μm particle size) provided of a Security Guard TM Ultra Cartridge (Phenomenex, Bologna, Italy). Chromatographic separation was obtained by using a mixture of formic acid in methanol 0.1% *v*/*v* (solvent A) and formic acid in H_2_O 0.1% *v*/*v* (solvent B) as eluent and developing a linear solvent gradient from 5 to 70% A within 18.5 min, at a flow rate 0.5 mL/min. The column and autosampler temperature were maintained at 35 and 4 °C, respectively. The acquisition of HR mass spectra was done in a scan range of *m/z* 150–1200 in ESI negative ionization mode, operating in full (70,000 resolution, 220 ms maximum injection time) and data dependent-MS/MS scan (17,500 resolution, 60 ms maximum injection time). Ionization parameters were optimized as previously reported [37]. Data were elaborated using the Xcalibur^TM^ software.

For the quantitative analysis of the major chemical constituents, three calibration curves were constructed using hesperidin, vitexin (apigenin 8-*C*-glucoside), and limonin as external standards for quantification of flavanon *O*-glycosides, flavone *C*-glucosides, and limonoids, respectively. Stock methanol solutions (1 mg/mL) were first prepared and then diluted by serial dilution to obtain solutions in triplicate at different concentrations in the following range: 0.00625–0.05 mg/mL for hesperidin and vitexin, 0.00156–0.0125 mg/mL for limonin. The calibration curves were constructed plotting concentrations with respect to the areas obtained by MS peak integration. The relation between variables was obtained by linear simple correlation (*R*^2^ = 0.9647 for hesperidin; *R*^2^ = 0.9895 for vitexin, and *R*^2^ = 0.9972 for limonin). Data were obtained by Microsoft^®^ Office Excel, and the amounts of components were finally expressed as mg/100 g ± standard deviation (SD) of original product.

### 2.7. Headspace-Solid Phase Microextraction (HS-SPME) Analysis

The volatile organic compounds (VOCs) of the fruits used for the manufacturing of the analysed products and of the two *Citrus*-based products were analysed by the headspace-solid phase microextraction (HS-SPME) method. All the samples (a slice including peel of each *Citrus* and 1 g of each *Citrus*-based product) were placed into a 50 mL glass flask, covered with an aluminum foil, and left to equilibrate for 30 min at room temperature. Thereafter, a sampling of 5 s for the fruits and 5 min for the marmalades was performed using a Supelco PDMS fiber (100 µm), preconditioned according to the manufacturer instructions. Once sampling was finished, the fiber was withdrawn into the needle and transferred to the injection port of the GC-MS system.

### 2.8. GC-MS Analyses

The gas chromatography-electron impact mass spectrometry (GC-EIMS) analyses were performed using an Agilent 7890B gas chromatograph (Agilent Technologies Inc., Santa Clara, CA, USA) equipped with an Agilent HP-5MS capillary column (30 m × 0.25 mm; coating thickness 0.25 μm) and an Agilent 5977B single quadrupole mass detector. The used analytical method was as follows: oven temperature rising from 60 to 240 °C at 3 °C/min; injector temperature of 220 °C; transfer line temperature of 240 °C; carrier gas helium of 1 mL/min. The mass spectra were acquired on full scan in a scan range of 30–300 *m/z* and a scan time of 1.0 s.

The peak identification was based on both a comparison of the retention times with those of pure samples, comparing their linear retention indices relative to the series of *n*-hydrocarbons (C8-C27), and a computer matching against commercial (NIST 14 and ADAMS 2007) and laboratory-developed mass spectra libraries built up from pure substances and components of commercial essential oils of known composition and MS literature data [47,48,49,50,51,52].

### 2.9. Sensorial Evaluation and Physicochemical Analysis

The sensorial evaluation of the Mixed Citrus and Lemon samples was performed at the Pharmacy Department of the University of Pisa, using a panel made up by 30 untrained panelists, comprising students, technicians, researchers, and professors, aged between 24 and 68 years. A 9-point hedonic scale, with 1 = dislike extremely and 9 = like extremely, was used to assess a set of attributes for each sample: colour, aroma, taste, flavour, texture, spreadability, and overall acceptability. The samples, three replicates for each one, were presented in a random pattern and a glass of water was provided to the panelists to rinse the mouth between each tasting, according to the Emelike and Akusu method [53]. The physicochemical analysis of the marmalades such as total sugars, titratable acidity (%), and sugar-acid ratio were evaluated. Total sugars (TS) were determined using a laboratory refractometer RL3 (Polkie Zaklady Optyczne, Warszawa, Poland) and expressed in °Brix [33]. The determination of titratable acid (TTA) was performed on 10 g of *Citrus*-based samples, homogenized in 100 mL of distilled water, and measured with 0.1 N NaOH with phenolphthalein-ethanol solution as an indicator. TTA content was expressed as % of citric acid [33]. All the experiments were performed in triplicate. Finally, the sugar-acid ratio was calculated.

### 2.10. Statistical Analysis

The *t*-test was carried out between the two samples (Mixed Citrus and Lemon), concerning the aroma composition (chemical compounds and classes) and the sensorial properties (colour, aroma, taste, flavour, texture, spreadability, and overall acceptability), while ANOVA test was carried out among all the samples for TPC and antioxidant assays. *p* < 0.05 was used to assess the significance of differences between means. The statistical analyses were performed using the JMP software package (SAS Institute, Cary, NC, USA).

## 3. Results

### 3.1. Total Polyphenol Content and Antioxidant Potential Evaluation

TPC and antioxidant power of the fruit pulps and peels and of the two *Citrus*-based products are reported in Table 3. Concerning the fresh fruits, the peels showed the highest content of polyphenols, even though statistically differences were observed. The mandarin peels sample was the richest one, showing a content of 68.24 ± 1.49 mg GAE/100 g of fresh weight (FW), followed by lemon peels (59.45 ± 1.23 mg GAE/100 g) and orange peels (41.21 ± 3.03 mg GAE/100 g). The fruit pulps, instead, were characterized by a TPC significantly lower than the peels, comprising between 13.47 ± 0.56 mg GAE/100 g in the orange and 19.24 ± 0.27 mg GAE/100 g in the lemon ones.

Concerning the *Citrus*-based products, both the Lemon and the Mixed Citrus samples were characterized by good amounts of polyphenols, even though they resulted significantly higher in the former sample, reaching a concentration of 24.28 ± 0.95 mg GAE in 100 g of product, than in the latter (15.79 ± 0.85 mg GAE/100 g). Similarly, both the antioxidant assays (DPPH and ABTS) evidenced the highest antioxidant activity of the peels, followed by the *Citrus* marmalades, while the pulp were the samples with the lowest antioxidant power. Concerning the marmalades, DPPH assay evidenced a significantly difference between the antioxidant power of Mixed Citrus and Lemon ones, resulting higher in the latter, with a lower IC_50_. The dose-response curves for DPPH radical scavenging capacity of all the samples are reported in Figures S1–S4 (Supplementary Materials). The ABTS assay, instead, showed a higher antiradicalic activity of the Lemon product than the Mixed Citrus one, but no statistically differences were evidenced.

### 3.2. Chemical Characterization and Amount of Bioactive Molecules

**Figure 1:**
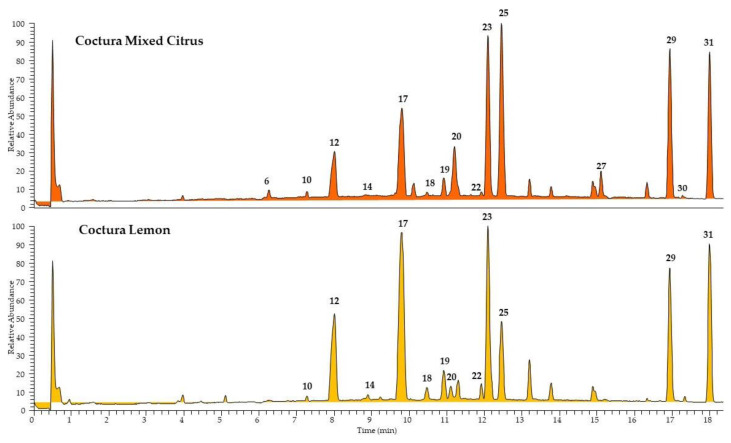
UHPLC-HR-ESI-MS/Orbitrap profiles, registered in negative ionization mode, of *n*-butanolic extracts of Lemon and Mixed Citrus products. Peak data are shown in Table 4.

The phytochemical profile of Lemon and Mixed Citrus Coctura^®^ samples, as well as pulp and peels of fresh *Citrus* fruits, was investigated in depth by means of UHPLC-HR-ESI-MS/Orbitrap, a highly sensitive technique useful for the identification of substances in complex mixtures. The LC-MS chromatograms obtained from the analyses of the two *Citrus*-based products are reported in Figure 1. From the qualitative point of view, the two samples showed a very similar composition, characterized by the presence of two major classes of components, flavonoids and triterpenoids. All compounds were identified based on their elution order, full and fragmentation MS data (Table 4) compared with the literature data. According to the literature, among flavonoids, several flavanone *O*-glycosides were tentatively identified: eriocitrin and/or its isomer neoericitrin (peak 17), narirutin and/or naringin (peak 20), and hesperidin and/or neohesperidin (peak 25), the most representative components in *Citrus* genus [21]. Since each couple of isomers differ for the sugar portion constituted by rutinose or its isomer neohesperidose, the correct identification it is not possible based only on MS data. However, eriocitrin, narirutin, and hesperidin are reported as the main component of *Citrus* juice, while neoeriocitrin, naringin, and neohesperidin are predominant in the peels [21]. In addition, four flavone *C*-glucosides, apigenin (peak 10) and diosmetin (peaks 14, 18, and 19) derivatives, were identified in both extracts by MS data [39]. In both the extracts, also three triterpenoids, belonging to the limonoid class, were identified and assigned as nomilinic acid glucoside (peak 23), limonin (peak 29), and nomilinic acid aglycone (31). The annotated compounds by MS data were previously isolated in *Citrus* fruits and identified by NMR techniques [54,55,56,57,58,59,60,61,62]. The fragmentation spectra herein obtained by Orbitrap MS/MS experiments are shown in Figures S5–S7 (Supplementary Materials). A minor compound, the limocitrin *O*-glucoside-3-hydrohy-3-methylglutaryl (peak 22) [63] was found in both *Citrus* marmalades. The Mixed Citrus product showed the presence of the terpenoid roseoside (peak 6) [64], the flavanone *O*-glycoside poncirin (peak 27), and a limonoid (peak 30) not detected in the Lemon product.

**Figure 2:**
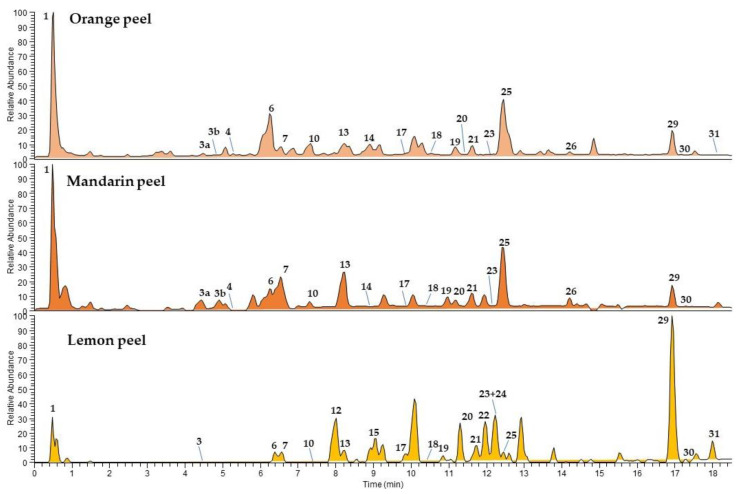
UHPLC-HR-ESI-MS/Orbitrap profiles, registered in negative ionization mode, of *n*-butanolic extracts of *Citrus* peels. Peak data are shown in Table 4.

**Figure 3:**
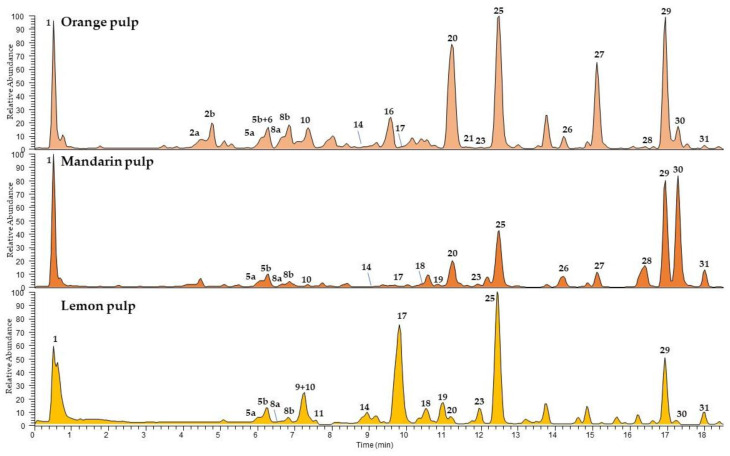
UHPLC-HR-ESI-MS/Orbitrap profiles, registered in negative ionization mode, of *n*-butanolic extracts of *Citrus* pulps. Peak data are shown in Table 4.

The chemical profiles of *Citrus* peel and pulp are showed in Figure 2 and Figure 3, respectively. Compared to Coctura products, the chemical composition of fresh fruits showed as major components the same flavanone *O*-glycosides and limonoids, with (neo)hesperidin (**25**) and limonin (**29**) well represented in all fruits, and (neo)eriocitrin (**17**) prominent in lemon pulp. All fruits used for their manufacturing were richer in several minor compounds, belonging to different classes such as hydroxycinnamic acids, including feruloyl (**4**, **5**, and **13**), coumaroyl (**2**, **3**, and **7**), and sinapoyl (**8**) derivatives in all analysed *Citrus*, the terpenoids roseoside (**6**) and ichangin (**28**) in mandarin and orange pulps. The phenylpropanoid xanthoxylin (**15**) and two methoxyflavonoids (**22** and **24**) were detected only in lemon peels. Several minor peaks remained unidentified.

**Figure 4:**
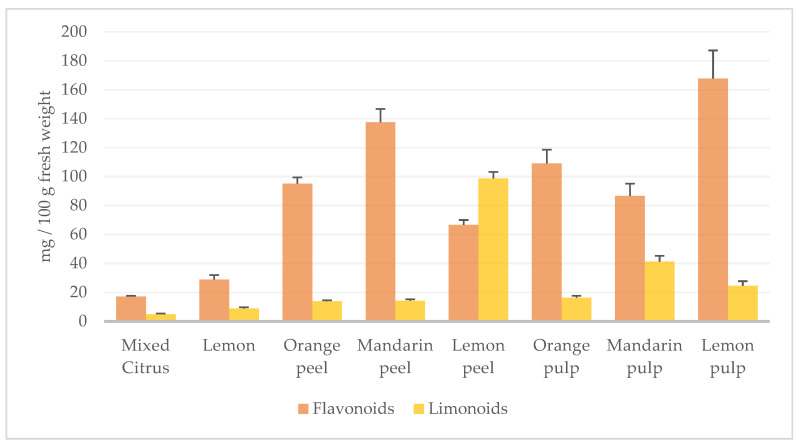
Distribution of flavonoids and limonoids in Coctura^®^ products (Mixed Citrus and Lemon) and fresh *Citrus* fruits.

Results of quantitative analysis (Table 5) showed that the Lemon sample was higher in both flavonoid and limonoid content than the Mixed Citrus one (28.9 ± 3.14 vs. 17.2 ± 0.45 and 8.85 ± 0.94 vs. 4.95 ± 0.41 mg/100 g of Coctura^®^ product, respectively). Among flavonoids, flavanone *O*-glycosides were more represented than flavones *C*-glucosides, with eritriocin the most abundant in both samples, but three times higher in the Lemon than in the Mixed Citrus, while the latter sample was richer in naringin and hesperidin. The three limonoids were found in comparable amount, with nomilinic acid glucoside a little bit higher in both samples. Quantitative analysis of fresh fruits (Table 5) showed that, despite a reduction in terms of both flavonoid and limonoid contents during the preparation process of marmalades, the relative composition of all samples was maintained. Moreover, (neo)eriocitrin (**17**) was the most represented flavonoid in Coctura Lemon as well as in pulp and peel of fresh lemon, while a lower content was observed in Coctura Mixed Citrus where a lesser quantity of lemon was used. Analogously, naringin (**25**) was higher in Coctura Mixed Citrus than in the Lemon one, due to its presence in mandarin and orange where the flavanone was detected in a good amount, especially in the peels. Limonoids resulted higher in Coctura Lemon as expected by the great content observed in lemon peel, the richest sample in this class of metabolites, mainly represented by limonin. All quantitative results are summarized in the chart in Figure 4.

### 3.3. Aroma Composition

The complete compositions of the headspaces of the analysed *Citrus*-based samples are reported in Table 6. Overall, 20 compounds were identified, covering the 100% of the whole chemical profiles.

The volatile organic compounds (VOCs) of both the *Citrus*-based samples were represented mainly by monoterpene hydrocarbons, resulting higher in Mixed Citrus (96.9%) than in Lemon (89.9%). Among these secondary metabolites, limonene was undoubtedly the main detected compound, as it covered 80.8 and 70.5% of the Mixed Citrus and Lemon sample HSs, respectively. γ-Terpinene was also detected in good percentages in both the product aroma profiles (Mixed Citrus: 10.4%; Lemon: 12.2%).

In addition, the volatilome of the Lemon sample was characterized by the presence of good percentage of oxygenated monoterpenes (9.7%), which instead were lower in the Mixed Citrus one (2.9%). This difference was attributable to the larger amount of 4-terpineol in the former sample (5.8%) than in the latter one (1.7%).

The marmalades showed a similar aroma profile to the fresh *Citrus*, whose chemical composition was reported in Table 7. A total of 20 compounds were identified in orange, mandarin, and lemon volatile profiles, represented mainly by monoterpene hydrocarbons (98.0 and 99.8% in mandarin and orange, respectively), as observed for the marmalade samples. Limonene was the most abundant compound in all the analysed fruits, particularly in orange, in which it accounted for 93.3% of the whole composition. Lemon and mandarin, instead, showed lower amounts of this compound (60.6% and 66.8%, respectively), while good percentages of other chemicals, in particular β-pinene (14.8%) and γ-terpinene (13.3%) were detected in noticeable amounts in the lemon aroma. However, γ-terpinene was the second most abundant component (18.9%) of the mandarin volatile emission.

### 3.4. Sensorial Properties and Physicochemical Analysis

**Figure 5:**
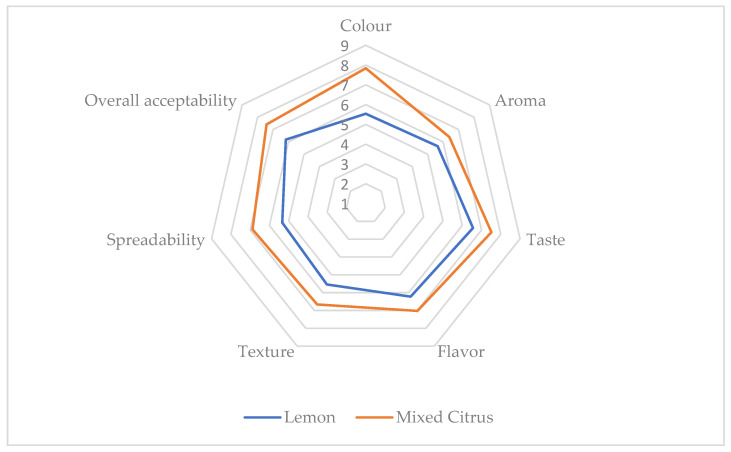
Radar graph of sensory analysis of Lemon and Mixed Citrus samples.

The average score and the radar graph of the different attributes perceived by the 30 untrained panelists who took part to the sensorial analysis of the Lemon and Mixed Citrus products are reported in Table 8 and Figure 5, respectively. Each attribute had a score between 1 and 9, according to the 9-point hedonic scale.

The scores for the sensory attributes of the Lemon sample ranged between 5.33 and 6.56. The spreadability was the parameter with the lowest score, followed by the colour (5.53), the texture (5.54), and the aroma (5.64). Flavour and taste, instead, scored more than 6 (6.22 and 6.56, respectively), as well as the overall acceptability (6.18), resulting in the “like slightly” area of the hedonic scale [74].

The Mixed Citrus scores were between 6.33 and 7.84. The highest value was achieved for the colour, followed by the taste (7.52), the overall acceptability (7.42), and the flavour (7.02). Scores between 7 and 8 corresponded to “like moderately” on the words version of the 9-point hedonic scale [74]. Scores under 7, instead, were obtained for the spreadability (6.88), the aroma (6.40), and the texture (6.33).

**Figure 6:**
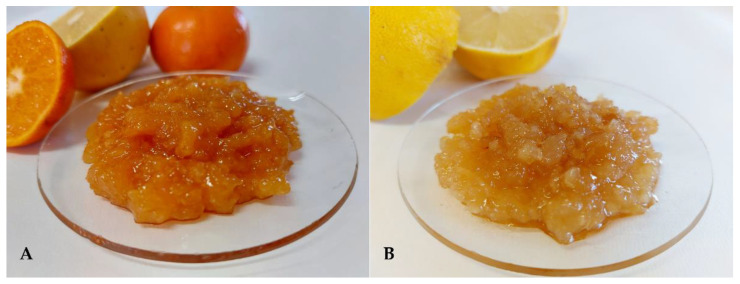
Coctura^®^ Mixed Citrus (**A**) and Lemon (**B**) products.

The total sugars and the titratable acidity of the Mixed Citrus and Lemon samples were 52.5 ± 0.436 and 57.6 ± 0.288 °Brix, and 1.25 ± 0.00% and 1.27 ± 0.00%, respectively. The sugar-acid ratio (TS/TTA) were 42.03 for Coctura^®^ Mixed Citrus and 45.43 for Coctura^®^ Lemon. As reported by Teixera et al. [33] higher SS/TA ratio indicates an improved taste due to a better balance between sugars and acids. As well evidenced by the radar graph, Mixed Citrus, despite its lower sugar-acid ratio, scored better than Lemon in the sensorial analysis for all the assessed attributes, evidencing the significant influence of the used *Citrus* species on the sensorial attributes, as well as on the overall acceptability of the final product. A picture of Mixed Citrus and Lemon samples is showed in Figure 6.

## 4. Discussion

In the present study, Coctura^®^ Lemon and Mixed Citrus products were evaluated and compared to the starting fresh fruits for their chemical and sensorial properties, in order to support the consumption of fruit-based products as source of health-promoting agents.

Both samples were found to be a source of phenolic compounds. The total phenolic content of the analysed *Citrus* products was lower than that reported by Rosa et al. [30] for both the orange marmalade (155.67 ± 48.2 mg GAE/100 g) and the mandarin/orange one (135.78 ± 46.74 mg GA/100 g), and by Rababah et al. [31], whose study highlighted a polyphenol content of 436.9 ± 28.2 mg GAE/100 g in an orange marmalade as well. Noticeable was the study conducted by Teixeira et al. [33], which investigated, among others, the polyphenolic content of marmalades produced with the addition of different percentages of orange peels during the formulation. The results evidenced a raising amount of phenols by increasing of the peels, reaching a maximum content of 12.73 ± 0.05 mg GAE/100 g, which, contrariwise to the previous mentioned studies, is lower than the phenolic compound content of the samples studied in the present work. To the best of our knowledge, however, this is the first study investigating the composition of marmalades made only from lemons and from a mixture of lemons, oranges, and mandarins. As a consequence, a comparison with the literature should be challenging.

Moreover, the amount of phenols in the analysed samples was accurately determined by UHPLC-MS quantitative analysis by using an external standard for each class of detected phytochemicals. To the best of our knowledge, this is the first paper reporting phytochemical analyses of *Citrus* marmalade by using this technique. The total flavonoid amount calculated by calibration curves agreed with results obtained spectrophotometrically. A total flavonoid content of 17.2 ± 0.45 and 28.9 ± 3.14 mg/100 g of Mixed Citrus and Lemon samples, respectively, was found. Compared to flavonoid content (35–147 mg/100 g) reported in edible part of orange [75], both *Citrus*-based products could be considered a good source of flavonoids.

Furthermore, according to the chemical data reported in the literature for fresh *Citrus* fruits and confirmed by quantitative analyses of starting fruits performed in this study, flavanone *O*-glycosides mostly contributed to the phenol composition of *Citrus*-based products. When only lemons were used, the final product showed a predominant amount of eriocitrin, followed by hesperidin and narirutin/naringin. This evidence is in agreement with data reported in the literature about lemon juice in which hesperidin and eriocitricin are the most significant flavanones, but with hesperidin usually the most abundant one, as observed for lemon pulp analysed in this study [18,19,76]. When lemons, sweet oranges, and mandarins were mixed for marmalade production, the ratio between the main flavanones was changed consequently. The total flavonoid content was found lower, with an increased amount of hesperidin, which resulted the main component, followed by eriocitricin. Similarly, the content of narirutin/naringin was higher in the Mixed Citrus sample. These findings are in agreement with results obtained by quantitative analysis of fresh fruits in this study and the literature data reporting mandarin and orange as a good source of hesperidin, followed by narirutin/naringin and eritriocin [18,19,76]. Lemon sample was also richer in flavone *C*-glucosides than Mixed Citrus, suggesting that lemon contributed greatly to the presence of these compounds in the product obtained with *Citrus* mixture. Similarly, limonoid content showed about 50% reduction when a mixture of *Citrus* fruits was used, as expected from the higher content of these components registered in lemon fruits, especially in lemon peel. According to Rababah et al. [31], a reduction in terms of both flavonoid and limonoid contents during the preparation process of marmalades was observed, probably due to a cell structure disruption during the fruit processing. However, the relative composition of all samples was maintained.

Probably related to their phenol content, the two *Citrus*-based products showed a good antioxidant potential, even though it was lower than that of the fresh *Citrus* fruit. Results agreed with Rababah et al. [31], who reported a reduction of the antioxidant power during the processing of a strawberry jam. The radical scavenging ability is related to many degenerative diseases and aging events, thus intake of antioxidant compounds is important for their prevention. Several epidemiological studies suggested a direct relation between flavonoid intake and decrease of cardiovascular diseases and other degenerative pathologies [77]. *Citrus* flavonoids, in particular hesperidin and naringin, were extensively studied for their antioxidant activity, which is related not only to their radical scavenging activity, but also to their involvement in the regulation of antioxidant gene expression. Hesperidin and naringin, as well as their aglycones, were found to play an important role in the prevention of pathological conditions related to oxidative stress and inflammation, also thanks to their ability to inhibit key enzymes involved in the inflammation response and to downregulate the production of pro-inflammatory cytokines [19]. The cardioprotective effect of hesperidin and naringin, as well as their potential in the prevention of atherosclerosis and cancer, is reported in many studies [19,21,22,78]. In addition, several studies highlighted a lipid-lowering effect exerted by flavanones, in particular naringin and hesperidin, in in vivo models after a diet supplemented with a flavanone nutritional dose [42,43,79]. More recently, the pharmacological properties of limonoids, as typical constituents of *Citrus* fruits previously considered undesirable bitter compounds, have been investigated in depth. Interestingly, limonin, the most abundant limonoid aglycone in *Citrus* seeds, was found to exert antitumor activity in in vivo studies, as well as antioxidant capacity, and hypocholesterolemic properties reducing apoB production in liver cells by >70% [23,24,80]. Limonin showed also antiviral activity towards human retroviruses [81]. Thus, both flavonoids and limonoids could play a role in exerting beneficial effects in human health.

Our chemical investigation showed the presence of two aglycones, limonin and nomilinic acid, and the glucoside of nomilinic acid only. Limonoid aglycones are usually more abundant in seeds and peels, while glucosides, soluble in water and alcohols, predominate in the fruit pulp. The limonoid amount found in Coctura^®^ products suggested a good balance of *Citrus* peels and fruits in the formulations.

The aroma profile of the Mixed Citrus and Lemon samples reflected the VOCs emitted from the analysed fresh fruits employed for their formulation, which in turn, are in line with the literature data investigating the chemical composition of the EOs obtained from the fruit peels of these *Citrus* [35,82,83], as these by-products were exploited as natural sealing agents, promoting the reduction of food waste and as a consequence the circularization of the supply chain. The flavour, as well as the EOs, of *Citrus* fruits have been widely investigated over the years. According to our findings on the aroma composition of *C. limon*, Settanni et al. [83] reported monoterpene hydrocarbons as the main class of the peels essential oil of *C. limon* cv. Femminello Santa Teresa, mainly represented by limonene, followed by γ-terpinene and β-pinene. Contrarywise, very low percentages of sesquiterpene hydrocarbons were found [83]. Accordingly, almost the whole aroma profile of the Lemon sample, produced using the same lemon cultivar, was represented by the same main chemical class, with limonene, responsible for a fresh, citrus-like scent [35,84], followed by γ-terpinene, perceived as fresh-herbaceous, bitter, and citrus [35,84], as the most representative constituents, while the sesquiterpenes, least volatile, were even absent. Conversely, very low percentages of β-pinene were revealed, probably due to its high volatility.

The aroma of the Mixed Citrus sample showed a predominance of monoterpene hydrocarbons as well, as these secondary metabolites were the most represented in all the three employed fruit species, as reported in Table 5. Limonene was evidenced to be the major constituent here too, followed by myrcene and sabinene, both found in the HSs of all three fruit samples, even though in greater percentages in *C. sinensin* and *C. limon*, rather than in *C. reticulata*. These findings corroborate with the literature data. Ascrizzi et al. [35], indeed, reported the predominance of this class in both the hydrodistilled and manually squeezed essential oils (EOs) of *C. sinensis* (L.) Osbeck. Moreover, the hydrodistilled EO showed good percentages of oxygenated monoterpenes, represented mainly by the alcohol linalool, associated to floral, green, and citrus hints [35,85], which was found also in the HS of the analysed orange fruit, although in very low percentages (0.2%). The EO of *C. reticulata* Blanco, despite the fact that it was characterised predominantly by a percentage of monoterpene hydrocarbons as the EOs of lemon and orange, showed higher amounts of the oxygenated form of these secondary metabolites, mainly represented by *cis*-limonene oxide, *cis*-*p*-mentha-2,8-dienol, citronellol, and carvone [86]. These components, however, were not detected either in the fresh mandarin orange nor in the analyses Mixed Citrus product, even though its aroma was constituted by almost 10% of the whole composition by oxygenated monoterpenes. The main detected chemicals belonging to this class, instead, were α-terpineol and 4-terpineol, responsible for coniferous, cold, and stale, and musty, dusty, and spicy flavours, respectively [84,87]. The presence of notable percentages of these molecules, almost absent in the fresh fruits, may be considered negative as they were degradation products of limonene and linalool as a consequence of heating treatment and storage [88]. Perez-Lopez et al. reported that α-terpineol may be a significant off-flavour in mandarin juice stored for extended time due to its high stability and evidenced the opportunity to measure its formation as a quality parameter [88]. Despite the good percentage of this compound in the aroma profile, the Mixed Citrus sample was considered of good quality during the sensorial analysis, even better than the Lemon one. This should make us reflect on the importance of combining chemical and sensorial analysis in the food industry. Sensory evaluation, indeed, is a key tool as it allows to take into consideration the human perception of a food, deriving from the combination of chemical and physical stimuli [89], permitting the development of new products able to meet the consumer requirements in terms of organoleptic properties [90]. The acceptance of a food product is strictly connected to its success on the marketplace and depends on the ability of the food to meet the consumers expectations and needs [91]. It is an hedonic measure, which could be affected by several factors, such as the sensory properties of the food product, the expectations of consumers, the culture, and the physiological conditions [26]. The sensory properties include the characters of a food which could be described using the human senses, as colour, aroma, taste, flavour, texture, and spreadability. As the sensory properties are able to influence the consumers’ choices, they should be considered key attributes exploitable by the manufacturers for the improvement and the differentiation of their food products [91].

## 5. Conclusions

The cultivation and consumption of *Citrus* fruits is dramatically increasing worldwide, due to the recognition of their high-value chemical constituents with many beneficial effects to the human health and prevention of chronic diseases. The consumer demand for health-promoting food products is consequently raised, thus targeted studies are needed to enrich the knowledge about chemical and biological properties, as well as quality and safety. The chemical, biological, and sensorial investigations of *Citrus*-based products performed in this study showed that the most representative and biological active components of *Citrus* fruits, such as flavanone *O*-glycosides and limonoids persist even after the transformation process, by conferring antioxidant potential to the final products. Despite a reduction in terms of both flavonoid and limonoid contents during the preparation process of marmalades, the relative composition of all samples was maintained. (Neo)eriocitrin was the most represented flavonoid in Coctura^®^ Lemon as well as in pulp and peel of fresh lemon, while a lower content was observed in Coctura^®^ Mixed Citrus where a lesser quantity of lemon was used. Analogously, naringin was higher in Coctura Mixed Citrus than in the Lemon one, due to its presence in mandarin and orange, where the flavanone was detected in a good amount, especially in the peels. Limonoids resulted higher in Coctura Lemon as expected by the great content observed in lemon peel, the richest sample in this class of metabolites, mainly represented by limonin. The quantitative estimation of flavonoids and limonoids in the peel of lemon, orange, and mandarin used in manufacturing of Coctura^®^ products evidenced peels as a good source of both these bioactive agents. The utilization of peels in the product formulation can be considered a good choice useful to reevaluate a waste material for the preparation of food from local vegetal sources with benefits for human health. Furthermore, the aroma constituents and sensorial properties endow an added value to the Coctura^®^ *Citrus* preparations.

## Figures and Tables

**Table 1:** Composition (%) of Coctura^®^ *Citrus* products.

Coctura^®^ Products	Fruits (Pulp and Peel)	Cane Sugar
Mixed Citrus	Sweet oranges 37.5%, Femminello Santa Teresa lemons 9.5%, Avana mandarins 8.5%	44%
Lemon	Femminello Santa Teresa lemons 55%	44%

**Table 2:** Extraction yield of *Citrus* samples.

	Extract Weight (g)	
Sample (15 g)	MeOH	*n*-BuOH
Coctura^®^ Mixed Citrus	5.78	0.16
Coctura^®^ Lemon	5.70	0.27
Orange peel	1.18	0.12
Mandarin peel	1.28	0.16
Lemon peel	0.95	0.16
Orange pulp	1.16	0.07
Mandarin pulp	1.14	0.09
Lemon pulp	0.79	0.23

**Table 3:** Total polyphenol content (TPC) and radical scavenging effects assessed by DPPH and ABTS assays of the fruit pulps and peels and the *Citrus*-based products (Mixed Citrus and Lemon).

	TPC (mg GAE/100 g)	DPPH IC_50_ (mg/mL)	ABTS (TEAC mM/100 g)
Lemon pulp	19.24 ± 0.27 ^DE^	0.70 ± 0.037 ^C^	1.85 ± 0.08 ^C^
Lemon peels	59.45 ± 1.23 ^B^	0.14 ± 0.008 ^D^	11.77 ± 2.30 ^A^
Mandarin pulp	18.45 ± 0.59 ^DE^	1.14 ± 0.068 ^A^	1.75 ± 0.01 ^C^
Mandarin peels	68.24 ± 1.49 ^A^	0.21 ± 0.001 ^D^	13.36 ± 0.52 ^A^
Orange pulp	13.47 ± 0.56 ^E^	0.81 ± 0.053 ^C^	1.57 ± 0.14 ^C^
Orange peels	41.21 ± 3.03 ^C^	0.20 ± 0.002 ^D^	6.93 ± 0.22 ^B^
Coctura^®^ Mixed Citrus	15.79 ± 0.85 ^E^	1.00 ± 0.011 ^B^	3.34 ± 0.42 ^BC^
Coctura^®^ Lemon	24.28 ± 0.95 ^D^	0.26 ± 0.003 ^D^	3.61 ± 0.17 ^BC^

The results are expressed as mg gallic acid equivalents (GAE)/100 g FW (n = 3) ± standard error for TPC, and as IC_50_ (mg/mL) (n = 3) ± standard error and mmoL Trolox equivalents (TAEC)/100 g FW (n = 3) ± standard error for DPPH and ABTS, respectively. The superscript uppercase letters (A–E) indicate statistically significant differences between the samples.

**Table 4:** Chromatographic and mass spectrometry data of compounds identified in the analysed samples: Coctura Mixed Citrus (A), Coctura Lemon (B), Orange peel (C), Mandarin peel (D), Lemon peel (E), Orange pulp (F), Mandarin pulp (G), Lemon pulp (H). Peak numbers correspond to those of Figure 1, Figure 2 and Figure 3.

Peak	Compound ^a^	*t*_R_ (min)	HR-[M − H]^−^ (*m/z*)	HR-MS/MS Product Ions (*m/z*) ^b^	Molecular Formula	Mass Error (ppm)	Extract	Ref.
	*Organic acid and derivatives*							
**1**	Citric acid	0.5	191.0193	173.01, **111.01**, 87.01, 85.03	C_6_H_8_O_7_	−2.25	C, D, E, F, G, H	[39]
**9**	Aconitic acid	7.3	173.0086	129.09, **111.01**, 85.03	C_6_H_6_O_6_	−3.23	H	
**11**	Citric acid derivative	7.7	247. 0821	185.08, 173.01, **111.01**, 87.01, 85.03	C_10_H_16_O_7_	−0.93	H	[65]
	*Phenolic acid*							
**2a, 2b**	*cis/trans* Coumaroyl acid glucoside	4.4, 4.8	325.0929	**145.03**, 117.03	C_15_H_18_O_8_	+0.03	F	[66,67]
**3a, 3b**	*cis/trans* Coumaroylisocitric acid	4.5	337.0567	191.01, 163.04, 129.02, 119.05, **85.03**	C_15_H_14_O_9_	+0.59	C, D, E	[65]
**4**	Feruloylisocitric acid	5.3	367.0670	193.05, 147.03, 134.04, **85.03**	C_16_H_16_O_10_	−0.27	C, D	[65]
**5a, 5b**	*cis/trans* Feruloyl glucoside	6.2, 6.3	355.1035	193.05, **175.04**	C_16_H_20_O_9_	+0.11	F, G, H	[66,67]
**7**	*p*-Coumaric acid	6.5	163.0396	**119.05**, 104.03	C_9_H_8_O_3_	−2.88	C, D, E	[66,67]
**8a, 8b**	*cis/trans* Sinapoyl glucoside	6.8	385.1142	**223.06**, 205.05, 119.01, 91.01	C_17_H_22_O_10_	+0.47	F, G, H	[66,67]
**12**	Dihydroxyhydrocinnamic acid hexoside	7.9	357.1190	**195.07**, 151.08, 121.03	C_16_H_22_O_9_	+0.31	A, B, E	[68]
**13**	Ferulic acid	8.2	193.0501	178.03, 149.06, **134.04**	C_10_H_10_O_4_	−2.75	C, D, E	[66,67]
	*Phenylpropanoid*							
**15**	Xanthoxylin	9.0	195.0658	177.05, **151.08**, 136.05, 121.03	C_10_H_12_O_4_	+2.46	E	[69]
	*Terpenoids*							
**6**	Roseoside	6.2	([M + HCOO]^−^ 431.1923)	**385.19**, 223.13, 205.12, 153.09	C_19_H_30_O_8_		A, C, D, E, F	[67]
**16**	Abscisic acid glucose ester	9.6	425.1819	**263.13**, 219.14, 151.08	C_21_H_30_O_9_	−0.45	F	[70]
**21**	Abscisic acid	11.6	263.1288	**219.14**, 204.11, 201.13, 151.08	C_15_H_20_O_4_	+0.30	C, D, E, F	[71]
	*Flavone C-glucosides*							
**10**	Vicenin-2 (apigenin 6,8-di-*C*-glucoside)	7.3	593.1515	503.12, 473.11, 383.08, **353.07**	C_27_H_30_O_15_	+0.51	A–H	[39,43]
**14**	Lucenin-2 4′-methyl ether (diosmetin 6,8-di-*C*-glucoside)	8.9	623.1618	503.12, 413.09, 383.08	C_28_H_32_O_16_	0.00	A, B, C, D, F, G, H	[39,43]
**18**	Diosmetin 8-*C*-glucoside or 6-*C*-glucoside	10.5	461.1091	371.08, **341.07**, 298.05	C_22_H_22_O_11_	+0.43	A, B, C, D, E, G, H	[43]
**19**	Diosmetin 8-*C*-glucoside or 6-*C*-glucoside	10.9	461.1091	371.08, 341.07, **298.05**	C_22_H_22_O_11_	+0.43	A, B, C, D, E, G, H	[43]
	*Flavanone O-glycosides*							
**17**	Eriocitrin/neoeriotricin	9.8	595.1672	**287.05**, 151.00, 135.04	C_27_H_32_O_15_	−0.34	A–H	[39,43]
**20**	Narirutin/naringin	11.2	579.1716 ([M + HCOO]^−^ 625.1772)	**271.06**, 151.00, 119.05	C_27_H_32_O_14_	−0.52	A–H	[39,43]
**25**	Hesperidin/neohesperidin	12.5	609.1824 ([M + HCOO]^−^ 645.1593)	**301.07**, 286.05, 151.00	C_28_H_34_O_15_	−0.16	A–H	[39,43]
**27**	Poncirin	15.1	593.1875 ([M + HCOO]^−^ 639.1933)	**285.08**, 270.05	C_28_H_34_O_14_	−0.13	A, F, G	[43]
	*Methoxyflavonoids*							
**22**	Limocitrin *O*-3-hdroxy-3-methylglutaryl (HMG)- glucoside-	12.0	651.1569	507.11, 345.06, **329.03**			A, B, E	[67]
**24**	Tetramethoxyflavonoid *O*-HMG-glucoside-	12.2	681.1673	537.12, 375.07, **359.04**, 345.02			E	[67]
	*Ciclopeptydes*							
**26**	Citrusin III	14.2	726.3830 ([M + HCOO]^−^ 772.3892)	696.37, 590.33, 119.05	C_36_H_53_N_7_O_9_	+0.28	C, D, F, G	[72]
	*Limonoids*							
**23**	Nomilinic acid glucoside	12.1	711.2872	651.27, 607.28, 370.36, **59.01**	C_34_H_48_O_16_	+0.28	A–H	[39]
**28**	Ichangin	16.4	487.1978 ([M + HCOO]^−^ 533.2028)	**469.19**, 411.15, 381.21, 147.08	C_26_H_32_O_9_	+0.90	F, G	[73]
**29**	Limonin	16.9	469.1861 ([M + HCOO]^−^ 515.1920)	321.11, 229.12, 199.11	C_26_H_30_O_8_	+0.43	A–H	[37,73]
**30**	Deacetyl nomilin/isobacunonic acid/limonol	17.3	471.2021 ([M + HCOO]^−^ 517.2078)	**471.20**, 453.19, 429.19, 303.72 ^c^	C_26_H_32_O_8_	−0.72	A, C, D, E, F, G, H	[62,73]
**31**	Nomilinic acid	18.0	531.2233	471.20, 427.21, 369.17, **59.01**	C_28_H_36_O_10_	−0.56	A, B, C, E, F, G, H	[37,73]

^a^ Compounds were tentatively identified based on comparison of mass spectra (full scan and fragmentation patterns) with literature. ^b^ The base ion peak is shown in bold. ^c^ Product ions generated by fragmentation of [M + HCOO]^−^ adduct.

**Table 5:** Amount of flavonoids and limonoids in Coctura^®^ products and fresh Citrus samples expressed as mg/100 g of fresh product.

	Amount (mg/100 g of Fresh Weight)							
	Coctura Products		Peel			Pulp		
Peak	Mixed Citrus	Lemon	Orange	Mandarin	Lemon	Orange	Mandarin	Lemon
Flavonoids								
**10**	0.729 ± 0.024	1.08 ± 0.13	7.57 ± 0.62	7.60 ± 0.63	1.38 ± 0.126	3.15 ± 0.35	1.26 ± 0.15	3.68 ± 0.53
**14**	0.431 ± 0.019	1.10 ± 0.13	0.456 ± 0.019	2.79 ± 0.34	nd	0.00390 ± 0.0024	0.163 ± 0.021	4.15 ± 0.60
**18**	0.527 ± 0.0084	1.36 ± 0.16	0.794 ± 0.028	2.48 ± 0.20	2.17 ± 0.043	nd	0.293 ± 0.027	2.59 ± 0.043
**19**	0.876 ± 0.015	2.13 ± 0.24	1.01 ± 0.11	6.82 ± 0.64	2.08 ± 0.19	nd	0.587 ± 0.063	4.90 ± 0.095
**17**	5.08 ± 0.081	16.4 ± 1.74	0.610 ± 0.034	0.706 ± 0.074	35.4 ± 1.5	0.0590 ± 0.067	0.266 ± 0.024	53.5 ± 5.6
**20**	2.10 ± 0.070	0.482 ± 0.055	6.44 ± 0.052	20.6 ± 2.3	0.734 ± 0.023	63.7 ± 6.1	34.2 ± 4.1	7.79 ± 1.3
**25**	7.48 ± 0.23	6.40 ± 0.69	78.2 ± 3.1	96.6 ± 4.9	24.9 ± 1.4	41.5 ± 3.0	49.9 ± 4.0	91.1 ± 11
Total	17.2 ± 0.45	28.9 ± 3.14	95.0 ± 4.4	138 ± 9.1	66.7 ± 3.4	109 ± 9.5	86.7 ± 8.4	168 ± 19
Limonoids								
**23**	1.79 ± 0.048	3.30 ± 0.37	0.467 ± 0.023	0.18 ± 0.016	1.09 ± 0.094	0.193 ± 0.020	0.587 ± 0.066	4.27 ± 0.59
**29**	1.66 ± 0.043	2.60 ± 0.26	13.2 ± 0.062	14.0 ± 1.1	86.5 ± 4.2	15.7 ± 1.4	35.4 ± 3.4	17.3 ± 2.1
**31**	1.50 ± 0.32	2.95 ± 0.31	0.182 ± 0.0088	nd	11.1 ± 0.34	0.392 ± 0.044	5.29 ± 0.44	3.06 ± 0.42
Total	4.95 ± 0.41	8.85 ± 0.94	13.9 ± 0.65	14.2 ± 1.1	98.6 ± 4.6	16.3 ± 1.4	41.3 ± 3.9	24.6 ± 3.1

nd = not detected.

**Table 6:** Complete composition of the headspaces of the Mixed Citrus and Lemon samples.

Compounds	l.r.i. ^1^	Class	Relative Abundance (%) ± Standard Deviation	
			Mixed Citrus	Lemon
α-Pinene	933	mh	0.5 ± 0.07	0.6 ± 0.11
Camphene	948	mh	0.1 ± 0.08 ^B^	0.3 ± 0.03 ^A^
β-Pinene	977	mh	0.5 ± 0.15	0.8 ± 0.14
Myrcene	991	mh	1.3 ± 0.09	1.2 ± 0.07
Octanal	1003	nt	0.1 ± 0.03 ^A^	- ^B^
α-Phellandrene	1006	mh	0.1 ± 0.02 ^B^	0.2 ± 0.01 ^A^
1,4-Cineole	1015	om	- ^B^	0.2 ± 0.04 ^A^
α-Terpinene	1017	mh	0.6 ± 0.08	0.5 ± 0.09
*p*-Cymene	1025	mh	0.9 ± 0.22	1.1 ± 0.15
Limonene	1029	mh	80.8 ± 1.76 ^A^	70.5 ± 3.29 ^B^
*(E)-*β-Ocimene	1047	mh	0.2 ± 0.17	0.2 ± 0.02
γ-Terpinene	1058	mh	10.4 ± 1.05	12.2 ± 0.66
Terpinolene	1089	mh	1.4 ± 0.06	2.2 ± 0.35
Linalool	1101	om	0.2 ± 0.03	0.3 ± 0.12
Nonanal	1105	nt	0.1 ± 0.07	0.2 ± 0.08
Fenchol	1114	om	0.2 ± 0.01	0.8 ± 0.33
Borneol	1165	om	- ^B^	0.3 ± 0.13 ^A^
4-Terpineol	1177	om	0.7 ± 0.09	2.2 ± 0.92
α-Terpineol	1191	om	1.7 ± 0.33	5.8 ± 2.35
Safranal	1201	ac	- ^B^	0.2 ± 0.07 ^A^
Monoterpene hydrocarbons (mh)			96.9 ± 0.48	89.9 ± 4.11
Oxygenated monoterpenes (om)			2.9 ± 0.47	9.7 ± 3.88
Apocarotenoids (ac)			- ^B^	0.2 ± 0.07 ^A^
Other non-terpene derivatives (nt)			0.2 ± 0.10	0.2 ± 0.16
Total identified (%)			100 ± 0.01	100 ± 0.02

^1^ Linear retention index on a HP 5-MS capillary column. For all the compounds and for the chemical classes, the superscript uppercase letters (A, B) indicate statistically significant differences between the samples. The statistically significance of the relative abundances was determined by the *t*-test, with *p* ≤ 0.05.

**Table 7:** Complete composition of the headspaces of fresh lemon, mandarin, and orange fruits.

Compounds	l.r.i. ^1^	Class.	Relative Abundance (%) ± Standard Deviation		
			Lemon	Mandarin	Orange
α-Thujene	926	mh	0.7 ± 0.00	1.0 ± 0.15	-
α-Pinene	933	mh	3.1 ± 0.11	2.3 ± 0.43	0.8 ± 0.03
Sabinene	973	mh	3.3 ± 0.08	0.3 ± 0.04	2.2 ± 0.11
β-Pinene	979	mh	14.8 ± 0.12	1.8 ± 0.27	0.2 ± 0.11
Myrcene	991	mh	1.9 ± 0.11	1.8 ± 0.38	2.7 ± 0.05
Octanal	1003	nt	-	0.6 ± 0.32	-
α-Phellandrene	1006	mh	0.1 ± 0.01	0.1 ± 0.03	-
α-Terpinene	1017	mh	0.7 ± 0.02	1.3 ± 0.03	-
*p*-Cymene	1024	mh	0.2 ± 0.09	3.3 ± 0.60	-
Limonene	1029	mh	60.6 ± 0.12	66.8 ± 2.29	93.3 ± 0.12
*(E)-*β-Ocimene	1047	mh	0.2 ± 0.01	-	-
γ-Terpinene	1058	mh	13.3 ± 0.01	18.9 ± 2.23	0.2 ± 0.17
Terpinolene	1089	mh	0.8 ± 0.03	1.1 ± 0.15	0.2 ± 0.01
Linalool	1101	om	-	0.2 ± 0.1	0.2 ± 0.02
4-Terpineol	1177	om	-	0.1 ± 0.06	-
α-Terpineol	1191	om	-	0.2 ± 0.12	-
β-Caryophyllene	1419	sh	0.1 ± 0.01	-	-
Methyl *N*-methylanthranilate	1408	nt	-	0.2 ± 0.16	-
*trans*-α-Bergamotene	1436	sh	0.1 ± 0.01	-	-
Valencene	1493	sh	-	-	0.2 ± 0.03
Monoterpene hydrocarbons (mh)			99.8 ± 0.01	98.0 ± 0.87	99.6 ± 0.05
Oxygenated monoterpenes (om)			-	0.7 ± 0.32	0.2 ± 0.02
Sesquiterpene hydrocarbons (sh)			0.2 ± 0.02	-	0.2 ± 0.03
Other non-terpene derivatives (nt)			-	1.3 ± 0.55	-
Total identified (%)			100.0 ± 0.01	100.0 ± 0.01	100.0 ± 0.01

^1^ Linear retention index on a HP 5-MS capillary column.

**Table 8:** Average scores and standard deviations for the different sensorial attributes of the sensorial analysis performed on the Lemon and Mixed Citrus products.

Sensorial Attributes	Mixed Citrus	Lemon
Colour	7.84 ± 0.14 ^A^	5.53 ± 0.14 ^B^
Aroma	6.40 ± 0.26 ^A^	5.64 ± 0.08 ^B^
Taste	7.52 ± 0.10 ^A^	6.56 ± 0.14 ^B^
Flavour	7.02 ± 0.25 ^A^	6.22 ± 0.12 ^B^
Texture	6.33 ± 0.27 ^A^	5.54 ± 0.15 ^B^
Spreadability	6.88 ± 0.30 ^A^	5.33 ± 0.20 ^B^
Overall acceptability	7.42 ± 0.16 ^A^	6.18 ± 0.05 ^B^

Superscript uppercase letters (A,B) indicates statistically significant differences between the samples for each attribute. The statistical significance of the relative abundances was established by the *t*-test, with *p* ≤ 0.05.

## Data Availability

The data presented in this study are available on request from the corresponding author.

## Supplementary Materials

The following are available online at https://www.mdpi.com/article/10.3390/foods11111550/s1: Figure S1. Dose-response curves for DPPH radical scavenging capacity of lemon pulp and peel *n*-butanolic extracts. The DPPH % of inhibition was plotted against concentration of the sample. The experiments were performed in triplicates. Figure S2. Dose-response curves for DPPH radical scavenging capacity of mandarin pulp and peel *n*-butanolic extracts. The DPPH % of inhibition was plotted against concentration of the sample. The experiments were performed in triplicates. Figure S3. Dose-response curves for DPPH radical scavenging capacity of orange pulp and peel *n*-butanolic extracst. The DPPH % of inhibition was plotted against concentration of the sample. The experiments were performed in triplicates. Figure S4. Dose-response curves for DPPH radical scavenging capacity of Coctura Lemon and Mixed Citrus *n*-butanolic extracts. The DPPH % of inhibition was plotted against concentration of the sample. The experiments were performed in triplicates. Figure S5. HR-ESI-MS/MS of flavone *C*-glucosides detected in *Citrus* fruits and Coctura^®^ products: vicenin-2 (peak 10), lucenin-2 4′-methyl ether (peak 14), diosmetin 8-*C*-glucoside and/or diosmetin 6-*C*-glucoside (peaks 18 and 19). Figure S6. HR-ESI-MS/MS of flavanones *O*-glycosides detected in *Citrus* fruits and Coctura^®^ products: eriocitrin/neoeriotricin (peak 17), narirutin/naringin (peak 20), hesperidin/neohesperidin (peak 25), poncirin (peak 27). Figure S7. HR-ESI-MS/MS of limonoids detected in *Citrus* fruits and Coctura^®^ products: nomilinic acid glucoside (peak 23), limonin (peak 29), deacetyl nomilin/isobacunonic acid/limonol (peak 30), nomilinic acid (peak 31).

## References

[1] Santeramo F.G., Carlucci D., De Devitiis B., Seccia A., Stasi A., Viscecchia R., Nardone G. (2018). Emerging trends in European food, diets and food industry. Food Res. Int..

[2] Aune D., Giovannucci E., Boffetta P., Fadnes L.T., Keum N., Norat T., Greenwood D.C., Riboli E., Vatten L.J., Tonstad S. (2017). Fruit and vegetable intake and the risk of cardiovascular disease, total cancer and all-cause mortality—A systematic review and dose-response meta-analysis of prospective studies. Int. J. Epidemiol..

[3] Miller V., Mente A., Dehghan M., Rangarajan S., Zhang X., Swaminathan S., Dagenais G., Gupta R., Mohan V., Lear S. (2017). Fruit, vegetable, and legume intake, and cardiovascular disease and deaths in 18 countries (PURE): A prospective cohort study. Lancet.

[4] Nöthlings U., Schulze M.B., Weikert C., Boeing H., van der Schouw Y.T., Bamia C., Benetou V., Lagiou P., Krogh V., Beulens J.W.J. (2008). Intake of Vegetables, Legumes, and Fruit, and Risk for All-Cause, Cardiovascular, and Cancer Mortality in a European Diabetic Population. J. Nutr..

[5] Wang X., Ouyang Y., Liu J., Zhu M., Zhao G., Bao W., Hu F.B. (2014). Fruit and vegetable consumption and mortality from all causes, cardiovascular disease, and cancer: Systematic review and dose-response meta-analysis of prospective cohort studies. BMJ.

[6] Yip C.S.C., Chan W., Fielding R. (2019). The Associations of Fruit and Vegetable Intakes with Burden of Diseases: A Systematic Review of Meta-Analyses. J. Acad. Nutr. Diet..

[7] Noncommunicable Diseases. https://www.who.int/news-room/fact-sheets/detail/noncommunicable-diseases.

[8] Healthy Diet. https://www.who.int/news-room/fact-sheets/detail/healthy-diet.

[9] Septembre-Malaterre A., Remize F., Poucheret P. (2018). Fruits and vegetables, as a source of nutritional compounds and phytochemicals: Changes in bioactive compounds during lactic fermentation. Food Res. Int..

[10] Pennington J.A.T., Fisher R.A. (2010). Food component profiles for fruit and vegetable subgroups. J. Food Compos. Anal..

[11] Scalbert A., Manach C., Morand C., Rémésy C., Jiménez L. (2005). Dietary Polyphenols and the Prevention of Diseases. Crit. Rev. Food Sci. Nutr..

[12] Han X., Shen T., Lou H. (2007). Dietary Polyphenols and Their Biological Significance. Int. J. Mol. Sci..

[13] Cases J., Romain C., Dallas C., Gerbi A., Cloarec M. (2015). Regular consumption of Fiit-ns, a polyphenol extract from fruit and vegetables frequently consumed within the Mediterranean diet, improves metabolic ageing of obese volunteers: A randomized, double-blind, parallel trial. Int. J. Food Sci. Nutr..

[14] Stoclet J.-C., Chataigneau T., Ndiaye M., Oak M.-H., El Bedoui J., Chataigneau M., Schini-Kerth V.B. (2004). Vascular protection by dietary polyphenols. Eur. J. Pharmacol..

[15] Pennington J.A.T., Fisher R.A. (2009). Classification of fruits and vegetables. J. Food Compos. Anal..

[16] FAO (2021). Citrus Fruit Statistical Compendium 2020. Rome.

[17] Liu Y., Heying E., Tanumihardjo S.A. (2012). History, Global Distribution, and Nutritional Importance of Citrus Fruits. Compr. Rev. Food Sci. Food Saf..

[18] Gattuso G., Barreca D., Gargiulli C., Leuzzi U., Caristi C. (2007). Flavonoid Composition of Citrus Juices. Molecules.

[19] Barreca D., Gattuso G., Bellocco E., Calderaro A., Trombetta D., Smeriglio A., Laganà G., Daglia M., Meneghini S., Nabavi S.M. (2017). Flavanones: Citrus phytochemical with health-promoting properties. BioFactors.

[20] Alam M.A., Subhan N., Rahman M.M., Uddin S.J., Reza H.M., Sarker S.D. (2014). Effect of Citrus Flavonoids, Naringin and Naringenin, on Metabolic Syndrome and Their Mechanisms of Action. Adv. Nutr..

[21] Tripoli E., La Guardia M., Giammanco S., Di Majo D., Giammanco M. (2007). Citrus flavonoids: Molecular structure, biological activity and nutritional properties: A review. Food Chem..

[22] Benavente-García O., Castillo J. (2008). Update on Uses and Properties of Citrus Flavonoids: New Findings in Anticancer, Cardiovascular, and Anti-inflammatory Activity. J. Agric. Food Chem..

[23] Manners G.D. (2007). Citrus Limonoids: Analysis, Bioactivity, and Biomedical Prospects. J. Agric. Food Chem..

[24] Gualdani R., Cavalluzzi M., Lentini G., Habtemariam S. (2016). The Chemistry and Pharmacology of Citrus Limonoids. Molecules.

[25] Sharma K., Mahato N., Cho M.H., Lee Y.R. (2017). Converting citrus wastes into value-added products: Economic and environmently friendly approaches. Nutrition.

[26] Estaji M., Mohammadi-Moghaddam T., Gholizade-Eshan L., Firoozzare A., Hooshmand-Dalir M.-A.-R. (2020). Physicochemical characteristics, sensory attributes, and antioxidant activity of marmalade prepared from black plum peel. Int. J. Food Prop..

[27] Castelló M.L., Heredia A., Domínguez E., Ortolá M.D., Tarrazó J. (2011). Influence of thermal treatment and storage on astringency and quality of a spreadable product from persimmon fruit. Food Chem..

[28] Mazur S.P., Nes A., Wold A.-B., Remberg S.F., Martinsen B.K., Aaby K. (2014). Effects of ripeness and cultivar on chemical composition of strawberry (*Fragaria × ananassa* Duch.) fruits and their suitability for jam production as a stable product at different storage temperatures. Food Chem..

[29] Güder A., Engİn M.S., Yolcu M., Gür M. (2014). Effect of Processing Temperature on the Chemical Composition and Antioxidant Activity of *Vaccinium Arctostaphylos* Fruit and Their Jam. J. Food Process. Preserv..

[30] Rosa A., Atzeri A., Deiana M., Scano P., Incani A., Piras C., Cesare Marincola F. (2015). Comparative antioxidant activity and 1H NMR profiling of Mediterranean fruit products. Food Res. Int..

[31] Rababah T.M., Al-Mahasneh M.A., Kilani I., Yang W., Alhamad M.N., Ereifej K., Al-u’datt M. (2011). Effect of jam processing and storage on total phenolics, antioxidant activity, and anthocyanins of different fruits. J. Sci. Food Agric..

[32] Donno D., Mellano M., Hassani S., De Biaggi M., Riondato I., Gamba G., Giacoma C., Beccaro G. (2018). Assessing Nutritional Traits and Phytochemical Composition of Artisan Jams Produced in Comoros Islands: Using Indigenous Fruits with High Health-Impact as an Example of Biodiversity Integration and Food Security in Rural Development. Molecules.

[33] Teixeira F., dos Santos B.A., Nunes G., Soares J.M., do Amaral L.A., de Souza G.H.O., de Resende J.T.V., Menegassi B., Rafacho B.P.M., Schwarz K. (2020). Addition of Orange Peel in Orange Jam: Evaluation of Sensory, Physicochemical, and Nutritional Characteristics. Molecules.

[34] Sicari V., Pellicanò T.M., Laganà V., Poiana M. (2018). Use of orange by-products (dry peel) as an alternative gelling agent for marmalade production: Evaluation of antioxidant activity and inhibition of HMF formation during different storage temperature. J. Food Process. Preserv..

[35] Ascrizzi R., Taglieri I., Sgherri C., Flamini G., Macaluso M., Sanmartin C., Venturi F., Quartacci M., Pistelli L., Zinnai A. (2018). Nutraceutical Oils Produced by Olives and Citrus Peel of Tuscany Varieties as Sources of Functional Ingredients. Molecules.

[36] Sanmartin C., Taglieri I., Macaluso M., Sgherri C., Ascrizzi R., Flamini G., Venturi F., Quartacci M.F., Luro F., Curk F. (2019). Cold-Pressing Olive Oil in the Presence of Cryomacerated Leaves of Olea or Citrus: Nutraceutical and Sensorial Features. Molecules.

[37] Flori L., Macaluso M., Taglieri I., Sanmartin C., Sgherri C., De Leo M., Ciccone V., Donnini S., Venturi F., Pistelli L. (2020). Development of Fortified Citrus Olive Oils: From Their Production to Their Nutraceutical Properties on the Cardiovascular System. Nutrients.

[38] Taglieri I., Sanmartin C., Venturi F., Macaluso M., Bianchi A., Sgherri C., Quartacci M.F., De Leo M., Pistelli L., Palla F. (2021). Bread Fortified with Cooked Purple Potato Flour and Citrus Albedo: An Evaluation of Its Compositional and Sensorial Properties. Foods.

[39] Da Pozzo E., De Leo M., Faraone I., Milella L., Cavallini C., Piragine E., Testai L., Calderone V., Pistelli L., Braca A. (2018). Antioxidant and Antisenescence Effects of Bergamot Juice. Oxid. Med. Cell. Longev..

[40] Flamini G., Pistelli L., Nardoni S., Ebani V., Zinnai A., Mancianti F., Ascrizzi R., Pistelli L. (2019). Essential Oil Composition and Biological Activity of “Pompia”, a Sardinian Citrus Ecotype. Molecules.

[41] Giovanelli S., Ciccarelli D., Giusti G., Mancianti F., Nardoni S., Pistelli L. (2020). Comparative assessment of volatiles in juices and essential oils from minor Citrus fruits (Rutaceae). Flavour Fragr. J..

[42] De Leo M., Piragine E., Pirone A., Braca A., Pistelli L., Calderone V., Miragliotta V., Testai L. (2020). Protective Effects of Bergamot (*Citrus bergamia* Risso & Poiteau) Juice in Rats Fed with High-Fat Diet. Planta Med..

[43] Testai L., De Leo M., Flori L., Polini B., Braca A., Nieri P., Pistelli L., Calderone V. (2021). Contribution of irisin pathway in protective effects of mandarin juice (*Citrus reticulata* Blanco) on metabolic syndrome in rats fed with high fat diet. Phyther. Res..

[44] Singleton V.L., Rossi J.A.J. (1965). Colorimetry to total phenolics with phosphomolybdic acid reagents. Am. J. Enol. Vinic..

[45] Brand-Williams W., Cuvelier M.E., Berset C. (1995). Use of a free radical method to evaluate antioxidant activity. LWT Food Sci. Technol..

[46] Re R., Pellegrini N., Proteggente A., Pannala A., Yang M., Rice-Evans C. (1999). Antioxidant activity applying an improved ABTS radical cation decolorization assay. Free Radic. Biol. Med..

[47] Adams R.P. (1995). Identification of Essential Oil Components by Gas Chromatography/Quadrupole Mass Spectroscopy.

[48] Davies N.W. (1990). Gas chromatographic retention indices of monoterpenes and sesquiterpenes on Methyl Silicon and Carbowax 20M phases. J. Chromatogr. A.

[49] Jennings W., Shibamoto T. (1982). Qualitative Analysis of Flavor and Fragrance Volatiles by Glass Capillary Gas Chromatography.

[50] Masada Y. (1976). Analysis of Essential Oils By Gas Chromatography And Mass Spectrometry.

[51] Stenhagen E., Abrahamsson S., McLafferty F.W. (1974). Registry of Mass Spectral Data.

[52] Swigar A.A., Silverstein R.M. (1981). Monoterpenes.

[53] Emelike N., Akusu O. (2019). Quality Attributes of Jams and Marmalades Produced from Some Selected Tropical Fruits. J. Food Process. Technol..

[54] Ye X., Cao D., Song F., Fan G., Wu F. (2016). Preparative separation of nine flavonoids from Pericarpium Citri Reticulatae by preparative-HPLC and HSCCC. Sep. Sci. Technol..

[55] Mencherini T., Campone L., Piccinelli A.L., García Mesa M., Sánchez D.M., Aquino R.P., Rastrelli L. (2013). HPLC-PDA-MS and NMR Characterization of a Hydroalcoholic Extract of *Citrus aurantium* L. var. amara Peel with Antiedematogenic Activity. J. Agric. Food Chem..

[56] Caristi C., Bellocco E., Panzera V., Toscano G., Vadalà R., Leuzzi U. (2003). Flavonoids Detection by HPLC-DAD-MS-MS in Lemon Juices from Sicilian Cultivars. J. Agric. Food Chem..

[57] Gentili B., Horowitz R.M. (1968). Flavonoids of citrus. IX. C-Glycosylflavones and a nuclear magnetic resonance method for differentiating 6- and 8-C-glycosyl isomers. J. Org. Chem..

[58] Masao N., Shintaro K., Sachiko E., Fumiko I. (1971). Flavonoids in Citrus and Related Genera. Agric. Biol. Chem..

[59] Dunlap W.J., Wender S.H. (1960). Purification and identification of flavanone glycosides in the peel of the sweet orange. Arch. Biochem. Biophys..

[60] Matsubara Y., Sawabe A., Iizuka Y. (1990). Structures of New Limonoid Glycosides in Lemon (*Citrus limon* Burm. f.) Peelings. Agric. Biol. Chem..

[61] Khalil A.T., Maatooq G.T., El Sayed K.A. (2003). Limonoids from *Citrus reticulata*. Z. Naturforsch. C.

[62] Bennett R.D., Hasegawa S., Herman Z. (1989). Glucosides of acidic limonoids in citrus. Phytochemistry.

[63] Rodríguez-Rivera M.P., Lugo-Cervantes E., Winterhalter P., Jerz G. (2014). Metabolite profiling of polyphenols in peels of *Citrus limetta* Risso by combination of preparative high-speed countercurrent chromatography and LC–ESI–MS/MS. Food Chem..

[64] Matsubara Y., Sawabe A., Iizuka Y., Okamoto K. (1988). Studies on physiologically active substances in citrus fruit peel. Part XII. Structures of monoterpenoid glycosides in orange (*Citrus sinensis* Osbeck.), hassaku (*Citrus hassaku* Hort.) and yuzu (*Citrus junos* Sier.) peelings. Yukagaku.

[65] Masike K., Mhlongo M.I., Mudau S.P., Nobela O., Ncube E.N., Tugizimana F., George M.J., Madala N.E. (2017). Highlighting mass spectrometric fragmentation differences and similarities between hydroxycinnamoyl-quinic acids and hydroxycinnamoyl-isocitric acids. Chem. Cent. J..

[66] Anagnostopoulou M.A., Kefalas P., Kokkalou E., Assimopoulou A.N., Papageorgiou V.P. (2005). Analysis of antioxidant compounds in sweet orange peel by HPLC-diode array detection-electrospray ionization mass spectrometry. Biomed. Chromatogr..

[67] Luo Y., Zeng W., Huang K.-E., Li D.-X., Chen W., Yu X.-Q., Ke X.-H. (2019). Discrimination of *Citrus reticulata* Blanco and *Citrus reticulata* ‘Chachi’ as well as the *Citrus reticulata* ‘Chachi’ within different storage years using ultra high performance liquid chromatography quadrupole/time-of-flight mass spect. J. Pharm. Biomed. Anal..

[68] Fayek N.M., Farag M.A., Abdel Monem A.R., Moussa M.Y., Abd-Elwahab S.M., El-Tanbouly N.D. (2019). Comparative Metabolite Profiling of Four Citrus Peel Cultivars via Ultra-Performance Liquid Chromatography Coupled with Quadrupole-Time-of-Flight-Mass Spectrometry and Multivariate Data Analyses. J. Chromatogr. Sci..

[69] Chen J., Shen Y., Chen C., Wan C. (2019). Inhibition of Key Citrus Postharvest Fungal Strains by Plant Extracts In Vitro and In Vivo: A Review. Plants.

[70] Xiong D.M., Liu Z., Chen H., Xue J.T., Yang Y., Chen C., Ye L.M. (2014). Profiling the dynamics of abscisic acid and ABA-glucose ester after using the glucosyltransferase UGT71C5 to mediate abscisic acid homeostasis in Arabidopsis thaliana by HPLC-ESI-MS/MS. J. Pharm. Anal..

[71] Mannina L., Sobolev A.P., Di Lorenzo A., Vista S., Tenore G.C., Daglia M. (2015). Chemical Composition of Different Botanical Origin Honeys Produced by Sicilian Black Honeybees (*Apis mellifera* ssp. *sicula*). J. Agric. Food Chem..

[72] Matsubara Y., Yusa T., Sawabe A., Iizuka Y., Takekuma S.I., Yoshida Y. (1991). Structures of New Cyclic Peptides in Young Unshiu (*Citrus unshiu* MARCOV.), Orange (*Citrus sinensis* OSBECK.) and Amanatsu (*Citrus natsudaidai*) Peelings. Agric. Biol. Chem..

[73] Avula B., Sagi S., Wang Y.-H., Wang M., Gafner S., Manthey J., Khan I. (2016). Liquid Chromatography-Electrospray Ionization Mass Spectrometry Analysis of Limonoids and Flavonoids in Seeds of Grapefruits, Other Citrus Species, and Dietary Supplements. Planta Med..

[74] Wichchukit S., O’Mahony M. (2015). The 9-point hedonic scale and hedonic ranking in food science: Some reappraisals and alternatives. J. Sci. Food Agric..

[75] Peterson J.J., Dwyer J.T., Beecher G.R., Bhagwat S.A., Gebhardt S.E., Haytowitz D.B., Holden J.M. (2006). Flavanones in oranges, tangerines (mandarins), tangors, and tangelos: A compilation and review of the data from the analytical literature. J. Food Compos. Anal..

[76] Khan M.K., Zill-E-Huma, Dangles O. (2014). A comprehensive review on flavanones, the major *citrus* polyphenols. J. Food Compos. Anal..

[77] Hertog M.G., Feskens E.J., Kromhout D., Hertog M.G., Hollman P.C., Hertog M.G., Katan M. (1993). Dietary antioxidant flavonoids and risk of coronary heart disease: The Zutphen Elderly Study. Lancet.

[78] Zou Z., Xi W., Hu Y., Nie C., Zhou Z. (2016). Antioxidant activity of Citrus fruits. Food Chem..

[79] Chanet A., Milenkovic D., Manach C., Mazur A., Morand C. (2012). Citrus Flavanones: What Is Their Role in Cardiovascular Protection?. J. Agric. Food Chem..

[80] Kurowska E.M., Banh C., Hasegawa S., Manners G.D., Berhow M.A., Hasegawa S., Manners G.D. (2000). Regulation of Apo B Production in HepG2 Cells by *Citrus* Limonoids. Citrus Limonoids: Functional Chemicals in Agriculture and Food.

[81] Balestrieri E., Pizzimenti F., Ferlazzo A., Giofrè S.V., Iannazzo D., Piperno A., Romeo R., Chiacchio M.A., Mastino A., Macchi B. (2011). Antiviral activity of seed extract from *Citrus bergamia* towards human retroviruses. Bioorg. Med. Chem..

[82] Tao N.G., Liu Y.J., Tang Y.F., Zhang J.H., Zhang M.L., Zeng H.Y. (2009). Essential oil composition and antimicrobial activity of *Citrus reticulata*. Chem. Nat. Compd..

[83] Settanni L., Randazzo W., Palazzolo E., Moschetti M., Aleo A., Guarrasi V., Mammina C., San Biagio P.L., Marra F.P., Moschetti G. (2014). Seasonal variations of antimicrobial activity and chemical composition of essential oils extracted from three *Citrus limon* L. Burm. cultivars. Nat. Prod. Res..

[84] Alvarez R.Q., Passaro C.C., Lara O.G., Londono J.L. (2011). Relationship between chromatographic profiling by HS- SPME and sensory quality of mandarin juices: Effect of squeeze technology. Procedia Food Sci..

[85] Obenland D., Collin S., Sievert J., Arpaia M.L. (2013). Mandarin flavor and aroma volatile composition are strongly influenced by holding temperature. Postharvest Biol. Technol..

[86] Espina L., Somolinos M., Lorán S., Conchello P., García D., Pagán R. (2011). Chemical composition of commercial citrus fruit essential oils and evaluation of their antimicrobial activity acting alone or in combined processes. Food Control.

[87] Sun J., Sun B., Ren F., Chen H., Zhang N., Zhang Y., Zhang H. (2021). Effects of storage conditions on the flavor stability of fried pepper (*Zanthoxylum bungeanum*) oil. Foods.

[88] Pérez-López A.J., Saura D., Lorente J., Carbonell-Barrachina Á.A. (2006). Limonene, linalool, α-terpineol, and terpinen-4-ol as quality control parameters in mandarin juice processing. Eur. Food Res. Technol..

[89] Pieracci Y., Ascrizzi R., Pistelli L., Flamini G. (2021). Comparison of the Chemical and Sensorial Evaluation of Dark Chocolate Bars. Appl. Sci..

[90] Mounjouenpou P., Ngono Eyenga S.N.N., Kamsu E.J., Bongseh Kari P., Ehabe E.E., Ndjouenkeu R. (2018). Effect of fortification with baobab (*Adansonia digitata* L.) pulp flour on sensorial acceptability and nutrient composition of rice cookies. Sci. Afr..

[91] Maina J.W. (2018). Analysis of the factors that determine food acceptability. Pharma Innov. J..

