# Supplementary material for: Phytochemical Characterization of Citrus-Based Products Supporting Their Antioxidant Effect and Sensory Quality
Source: Foods. 2022 May 25;11(11):1550. doi: 10.3390/foods11111550 (PMC9180594; doi:10.3390/foods11111550)
Supplement: Supplementary file 1 [file foods-11-01550-s001.zip › foods-1725770-supplementary.pdf]

foods

MDPI

Article

Phytochemical characterization of *Citrus*-based products supporting their antioxidant effect and sensory quality

Ylenia Pieracci [1], Laura Pistelli [2,3,4], Massimiliano Cecchi [5], Luisa Pistelli [1,3,4,*] and Marinella De Leo [1,3,4,*]

[1] Department of Pharmacy, University of Pisa, Via Bonanno Pisano 33, 56126 Pisa, Italy;
ylenia.pieracci@phd.unipi.it
[2] Department of Agriculture Food Environment, University of Pisa, Via del Borghetto 80, 56124 Pisa, Italy;
laura.pistelli@unipi.it
[3] IInterdepartmental Research Center, Nutraceuticals and Food for Health, University of Pisa,
Via del Borghetto 80, 56124 Pisa, Italy
[4] CISUP, Centre for Instrumentation Sharing, University of Pisa, Lungarno Pacinotti 43, 56126 Pisa, Italy
[5] Scientific Consultant, Via Novelli detto Yambo 19, 56124 Pisa, Italy; phdcecchi@gmail.com
* Correspondence: marinella.deleo@unipi.it

Supplementary Materials

Table of Contents

Figure S1. Dose-response curves for DPPH radical scavenging capacity of lemon pulp and peel *n*-butanolic extracts. The DPPH % of inhibition was plotted against concentration of the sample. The experiments were performed in triplicates.

Figure S2. Dose-response curves for DPPH radical scavenging capacity of mandarin pulp and peel *n*-butanolic extracts. The DPPH % of inhibition was plotted against concentration of the sample. The experiments were performed in triplicates.

Figure S3. Dose-response curves for DPPH radical scavenging capacity of orange pulp and peel *n*-butanolic extracst. The DPPH % of inhibition was plotted against concentration of the sample. The experiments were performed in triplicates.

Figure S4. Dose-response curves for DPPH radical scavenging capacity of Coctura Lemon and Mixed Citrus *n*-butanolic extracts. The DPPH % of inhibition was plotted against concentration of the sample. The experiments were performed in triplicates.

Figure S5. HR-ESI-MS/MS of flavone *C*-glucosides detected in *Citrus* fruits and Coctura® products: vicenin-2 (peak 10), lucenin-2 4′-methyl ether (peak 14), diosmetin 8-*C*-glucoside and/or diosmetin 6-*C*-glucoside (peaks 18 and 19).

Figure S6. HR-ESI-MS/MS of flavanones *O*-glycosides detected in *Citrus* fruits and Coctura® products: eriocitrin/neoeriotricin (peak 17), narirutin/naringin (peak 20), hesperidin/neohesperidin (peak 25), poncirin (peak 27).

Figure S7. HR-ESI-MS/MS of limonoids detected in *Citrus* fruits and Coctura® products: nomilinic acid glucoside (peak 23), limonin (peak 29), deacetyl nomilin/isobacunonic acid/limonol (peak 30), nomilinic acid (peak 31).

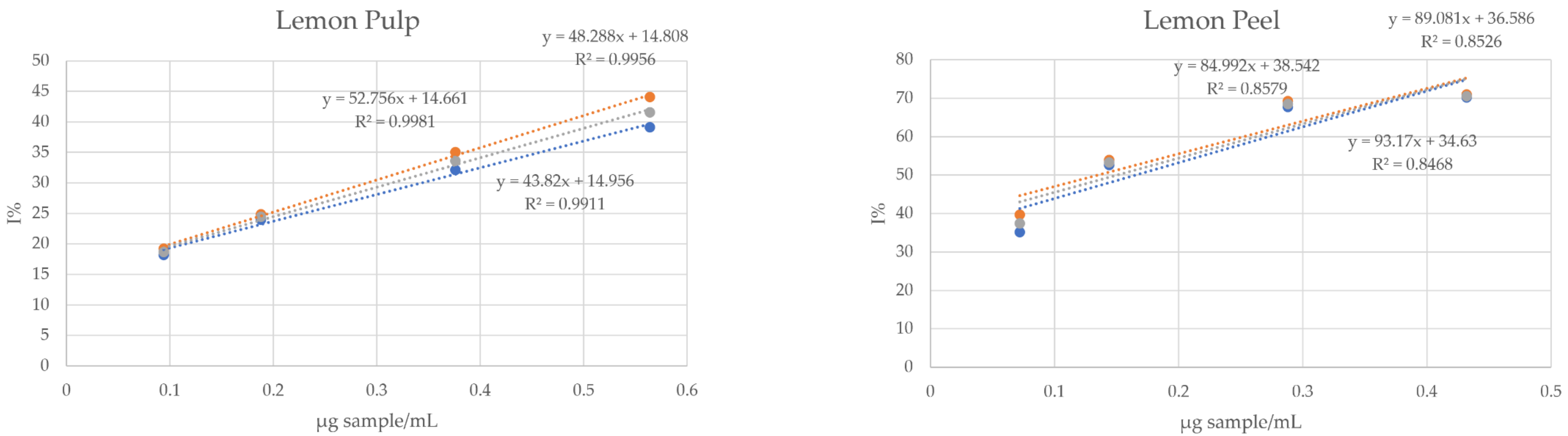

Figure S1. Dose-response curves for DPPH radical scavenging capacity of lemon pulp and peel *n*-butanolic extracts. The DPPH % of inhibition was plotted against concentration of the sample. The experiments were performed in triplicates.

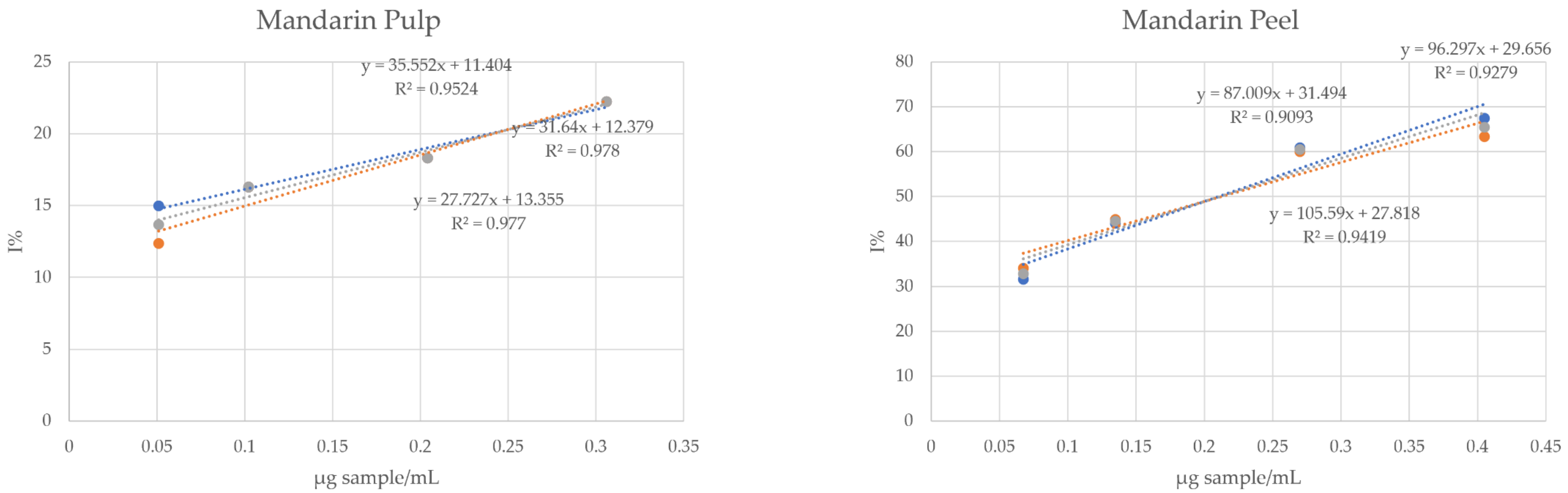

Figure S2. Dose-response curves for DPPH radical scavenging capacity of mandarin pulp and peel *n*-butanolic extracts. The DPPH % of inhibition was plotted against concentration of the sample. The experiments were performed in triplicates.

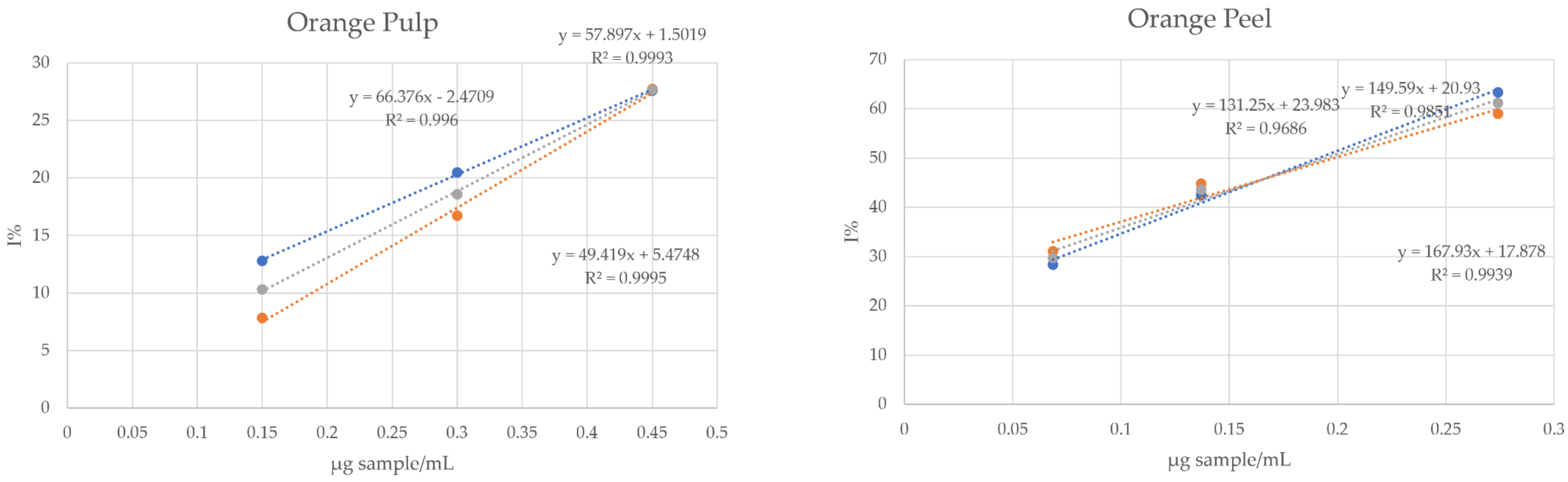

Figure S3. Dose-response curves for DPPH radical scavenging capacity of orange pulp and peel *n*-butanolic extracst. The DPPH % of inhibition was plotted against concentration of the sample. The experiments were performed in triplicates.

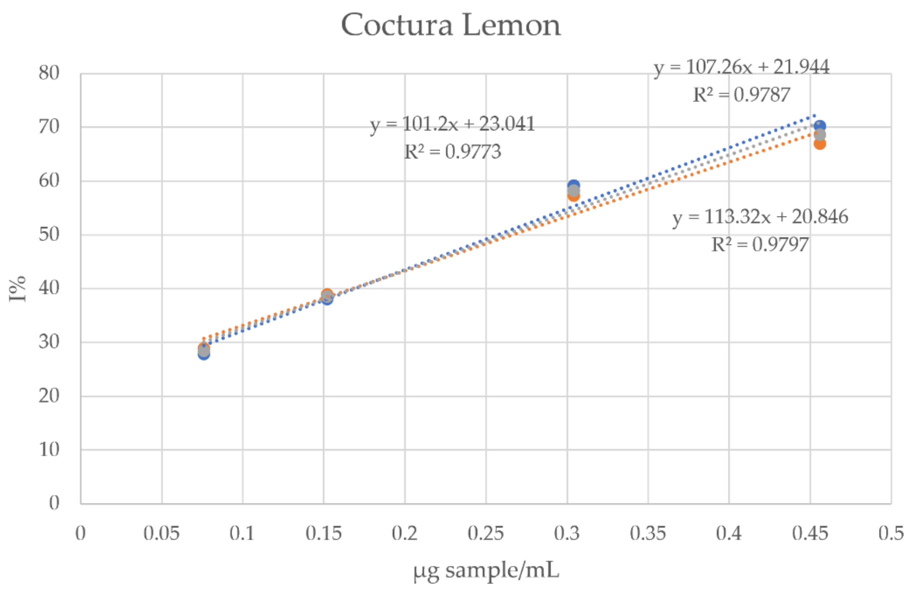

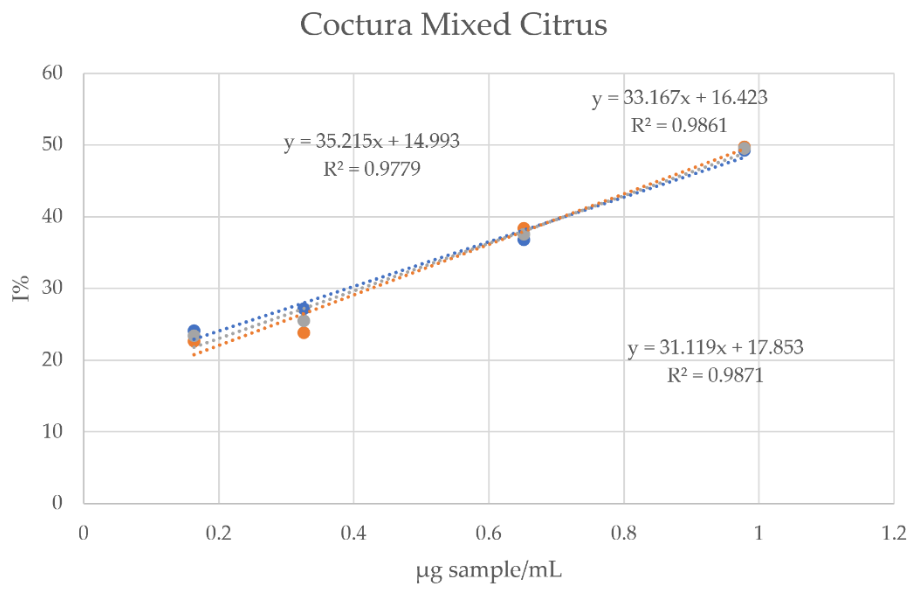

Figure S4. Dose-response curves for DPPH radical scavenging capacity of Coctura Lemon and Mixed Citrus *n*-butanolic extracts. The DPPH % of inhibition was plotted against concentration of the sample. The experiments were performed in triplicates.

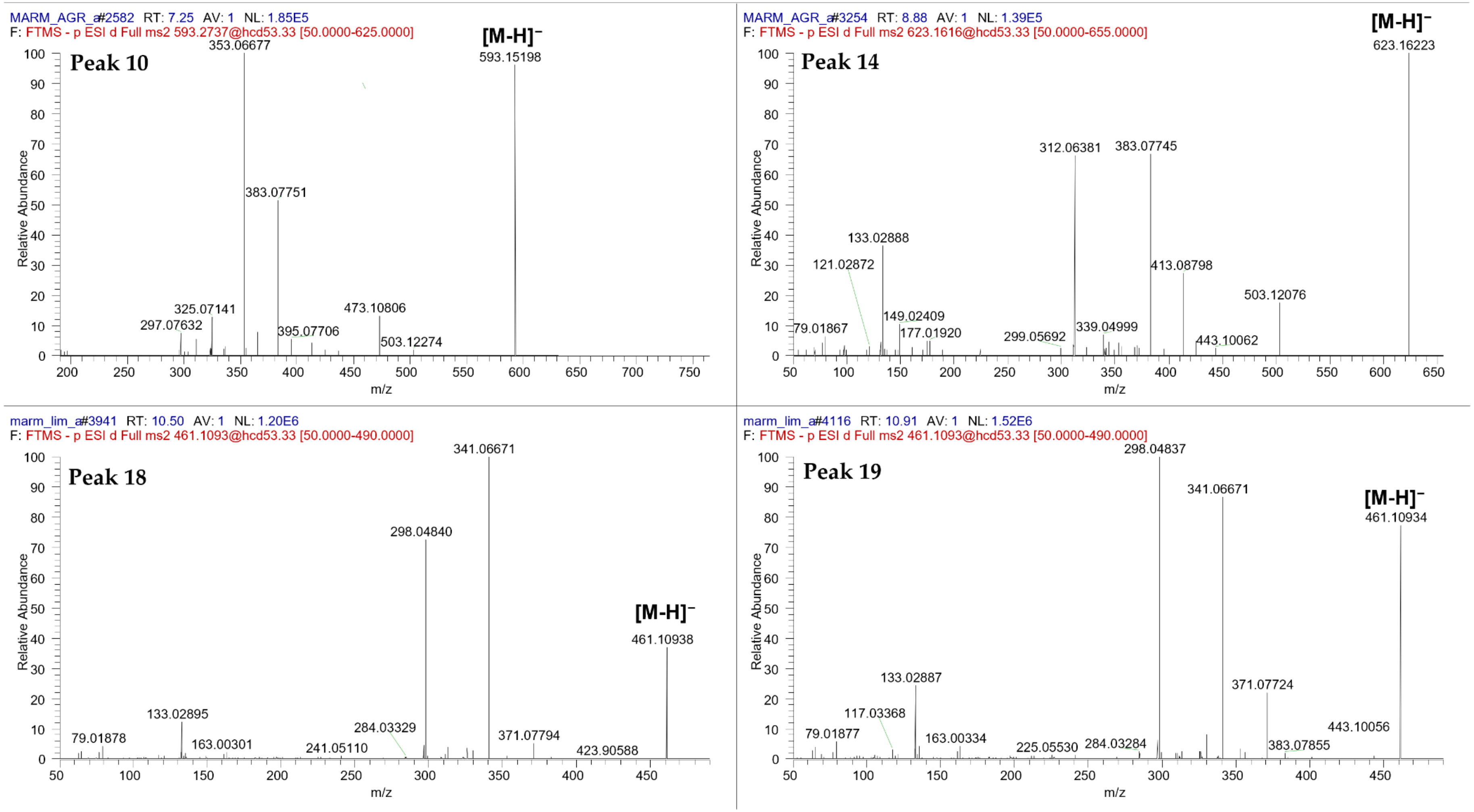

Figure S5. HR-ESI-MS/MS of flavone *C*-glucosides detected in *Citrus* fruits and Coctura® products: vicenin-2 (peak 10), lucenin-2 4′-methyl ether (peak 14), diosmetin 8-*C*-glucoside and/or diosmetin 6-*C*-glucoside (peaks 18 and 19).

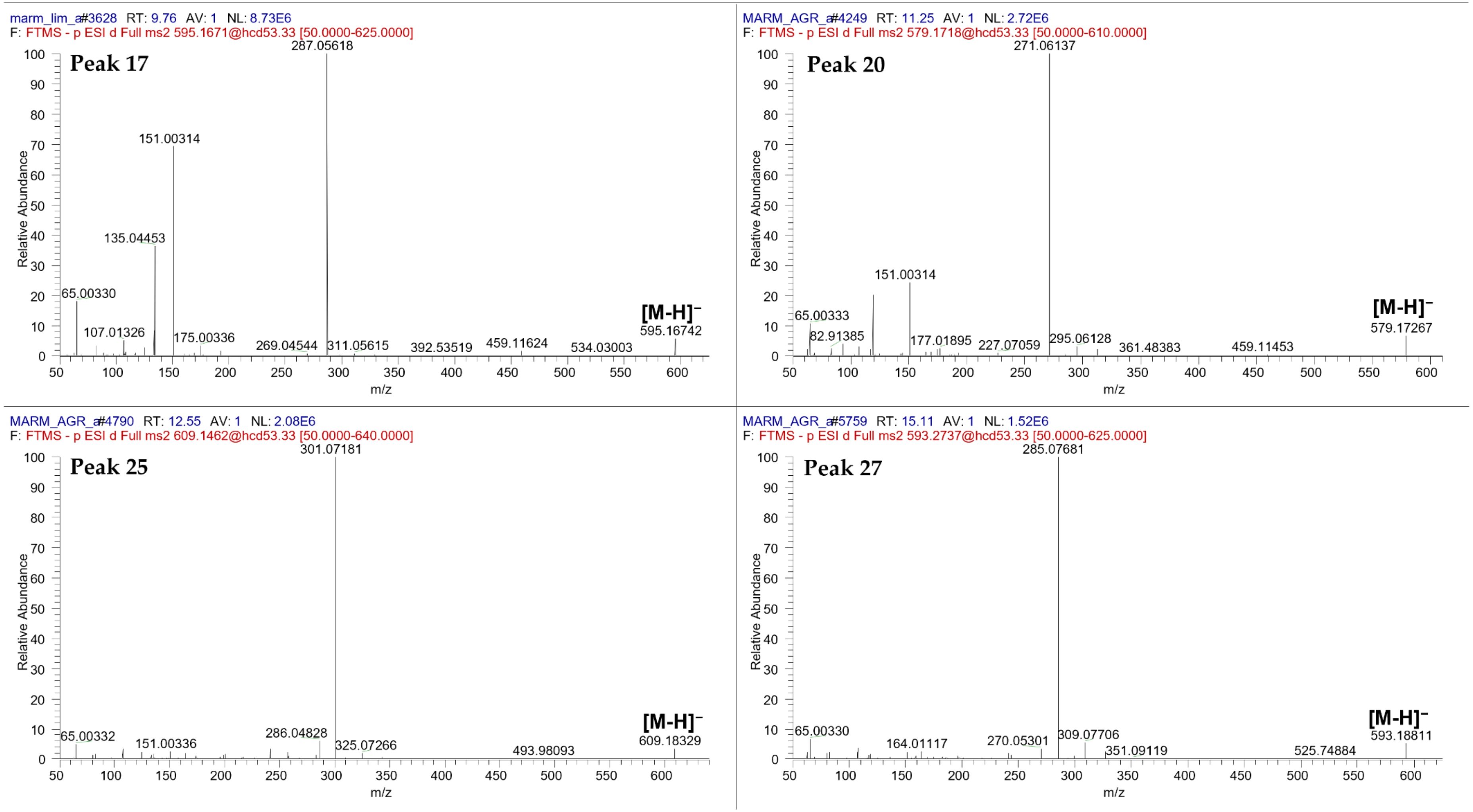

Figure S6. HR-ESI-MS/MS of flavanones *O*-glycosides detected in *Citrus* fruits and Coctura® products: eriocitrin/neoeriotricin (peak 17), narirutin/naringin (peak 20), hesperidin/neohesperidin (peak 25), poncirin (peak 27).

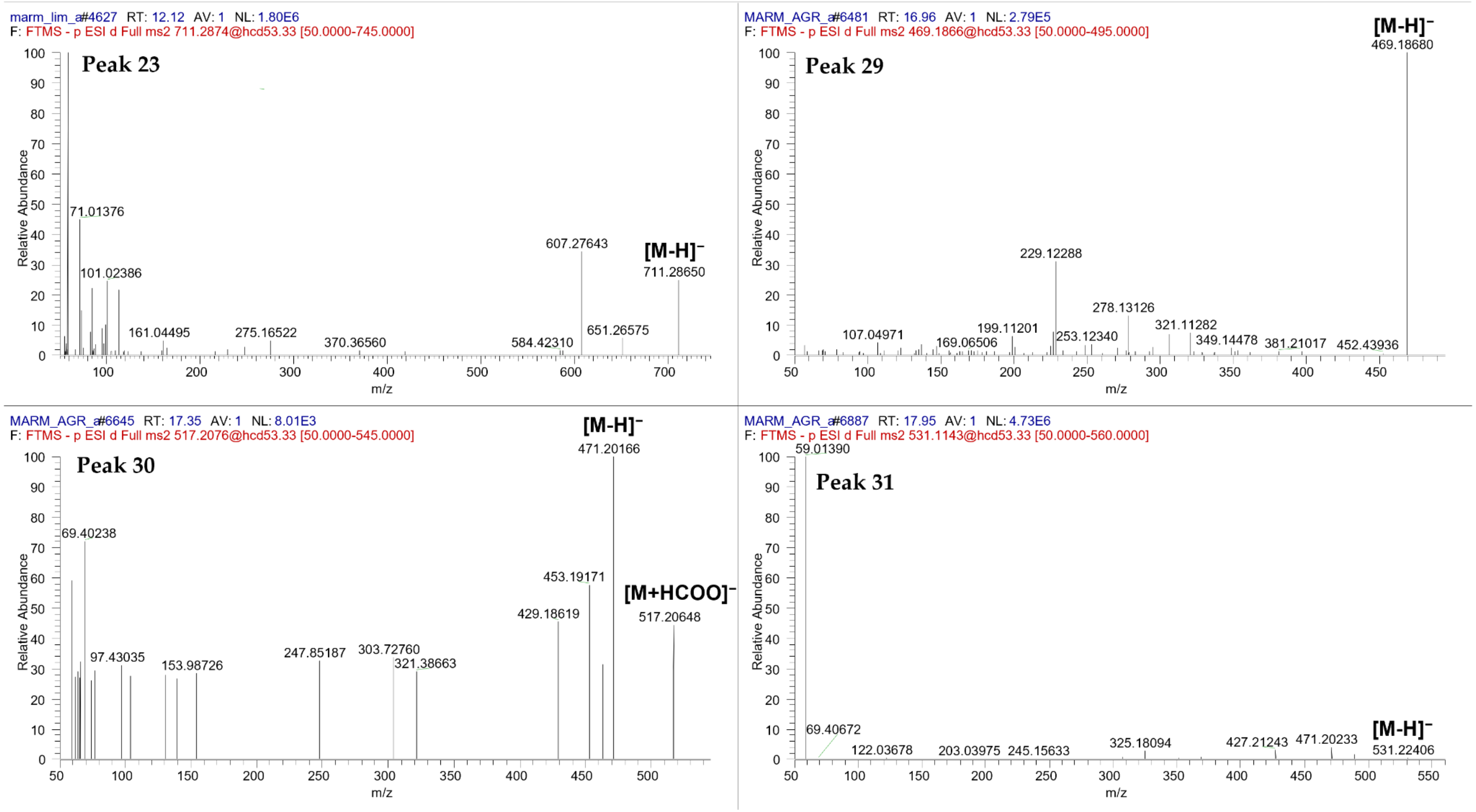

Figure S7. HR-ESI-MS/MS of limonoids detected in *Citrus* fruits and Coctura® products: nomilinic acid glucoside (peak 23), limonin (peak 29), deacetyl nomilin/isobacunonic acid/limonol (peak 30), nomilinic acid (peak 31).
